# Supplementary material for: Discovery of 1-Benzhydryl-Piperazine-Based HDAC Inhibitors with Anti-Breast Cancer Activity: Synthesis, Molecular Modeling, In Vitro and In Vivo Biological Evaluation
Source: Pharmaceutics. 2022 Nov 25;14(12):2600. doi: 10.3390/pharmaceutics14122600 (PMC9785542; doi:10.3390/pharmaceutics14122600)
Supplement: Supplementary file 1 [file pharmaceutics-14-02600-s001.zip › pharmaceutics-2038114-supplementary.pdf]

Supporting Information

Discovery of 1-Benzhydryl-Piperazine-Based HDAC Inhibitors with Anti-Breast Cancer Activity: Synthesis, Molecular Modeling, In Vitro and In Vivo Biological Evaluation

Dusan Ruzic [1], Bernhard Ellinger [2,3], Nemanja Djokovic [1], Juan F. Santibanez [4,5], Sheraz Gul [2,3], Milan Beljkas [1], Ana Djuric [6], Arasu Ganesan [7], Aleksandar Pavic [8], Tatjana Srdic-Rajic [6], Milos Petkovic [9,*] and Katarina Nikolic [1,*]

1 Department of Pharmaceutical Chemistry, Faculty of Pharmacy, University of Belgrade, Vojvode Stepe 450, 11221 Belgrade, Serbia
2 Fraunhofer Institute for Translational Medicine and Pharmacology (ITMP), 22525 Hamburg, Germany
3 Fraunhofer Cluster of Excellence for Immune-Mediated Diseases (CIMD), 22525 Hamburg, Germany
4 Group for Molecular Oncology, Institute for Medical Research, University of Belgrade, Dr. Subotića 4, 11129 Belgrade, Serbia
5 Centro Integrativo de Biología y Química Aplicada, Universidad Bernardo O'Higgins, Santiago 8370993, Chile
6 Department of Experimental Oncology, Institute for Oncology and Radiology of Serbia, Pasterova 14, 11000 Belgrade, Serbia
7 School of Pharmacy, University of East Anglia, Norwich Research Park, Norwich NR4 7TJ, UK
8 Institute of Molecular Genetics and Genetic Engineering, University of Belgrade, Vojvode Stepe 444a, 11000 Belgrade, Serbia
9 Department of Organic Chemistry, Faculty of Pharmacy, University of Belgrade, Vojvode Stepe 450, 11221 Belgrade, Serbia
* Correspondence: milosp@pharmacy.bg.ac.rs (M.P.); katarina.nikolic@pharmacy.bg.ac.rs (K.N.)

Table of contents

Table S1. The overview of specific HDAC roles in breast cancer cell migration, invasiveness and angiogenesis. 3

^{1}H and ^{13}C NMR spectra of the final compounds 2b to 9b. 4

LC-ESI (+) HRMS (ToF) mass spectra for compounds 6b – 9b in a range (m/z 100–1000) 12

Note S1. Molecular dynamics simulations 13

Figure S1. HDAC inhibition profiles of 6b (A), 7b (B), 8b (C) and 9b (D). 14

Figure S2. Root-Mean-Square-Deviation (RMSD) analysis of obtained trajectories. 15

Figure S3. Predicted binding mode of 6b and interaction of CAP group with L2 pocket of HDAC6. 16

Figure S4. Predicted binding mode of 9b and interaction of CAP group with L2 pocket of HDAC6. 17

Figure S5. Predicted binding mode of 7b and interaction of CAP group with L1 pocket of HDAC6. 18

Figure S6. Predicted binding mode of 8b and interaction of CAP group with L1 and L2 pockets of HDAC6. 19

Figure S7. Pairwise comparison of predicted binding modes of 6b (green sticks) and 9b (magenta sticks) in interaction with HDAC6 (gray cartoon). 20

Figure S8. Cell viability data generated after treating MDA-MB-231 cells (A) and MCF-7 cells (B) for 48 h with synthesized inhibitors. 21

Figure S9. Flow cytometry Annexin V-FITC/7-AAD staining of early and late apoptotic cell population at 5 μM 8b treatment of MDA-MB-231 cells. 22

Figure S10. The HDAC6 inhibitor 9b reduces MCF-7 cell migration. 23

Table S2. Lethal and teratogenic effects observed in zebrafish (*Danio rerio*) embryos at different hours post fertilization (hpf)... 24

References 25

Table S1. The overview of specific HDAC roles in breast cancer cell migration, invasiveness and angiogenesis.

HDAC isoform	Anti-migratory activity	Anti-invasive activity	Anti-angiogenic activity
**HDAC1**	• MDA-MB-231 and SkBr3 cells (Tang et al., 2017, p. 8)<br>• MCF7 (Palma et al., 2016)	• MDA-MB-231 and MCF-7 cells (Park et al., 2011)	• MDA-MB-468 cells (Entinostat) (Srivastava et al., 2010)
**HDAC2**	• MDA-MB-231 cells (VPA) (Zhang et al., 2012)	• MDA-MB-231 (Roy et al., 2014)	not found
**HDAC3**	• MDA-MB-231 cells (increased migration by HDAC3 depletion) (Kim et al., 2010) – HDAC3 specifically antagonizes the migration of MDA-MB-231 cells through the downregulation of CREB3-mediated CXCR4 expression	• MDA-MB-231 cells (increased invasivness by HDAC3 depletion) (Kim et al., 2010) – overexpression of HDAC3 decreased invasiveness of MDA-MB-231	• MDA-MB-468 cells (Entinostat) (Srivastava et al., 2010)<br>• increases vascular permeability by suppressing Robo4 expression in endothelial cells (Kashio et al., 2021)
**HDAC4**	• MDA-MB-231 cells (Hsieh et al., 2014)	• MDA-MB-231 cells (Hsieh et al., 2014)	• indirectly, inhibition of the proliferation and migration of vascular smooth muscle cells (Zheng et al., 2019, p. 1)
**HDAC5**	• MDA-MB-231 and Hs-578T cells (Hsieh et al., 2015; Li et al., 2016)	• MDA-MB-231 and Hs-578T cells (Hsieh et al., 2015; Li et al., 2016)	• negative regulator of angiogenesis in vitro and in vivo (Urbich et al., 2009)
**HDAC6**	• Endothelial cell migration (Kaluza et al., 2011)	• MDA-MB-231 and MCF-7 cells (Park et al., 2011; Rey et al., 2011)	• Zebrafish model (Kaluza et al., 2011)
**HDAC7**	not found	not found	• Endothelial cells migration (Mottet et al., 2007)
**HDAC8**	• MDA-MB-231 cells (An et al., 2019, 2020)	• MDA-MB-231 and MCF-7 cells (Park et al., 2011)	not found
**HDAC9**	• MCF-7 and BT474 (Huang et al., 2018)	• MDA-MB-231 cells (Salgado et al., 2018)	• MDA-MB-231 cells (Salgado et al., 2018)
**HDAC10**	not found	not found	• Endothelial cells (Duan et al., 2017)
**HDAC11**	• Negative control of migration (Leslie et al., 2019)	• Negative control of invasion (Yi et al., 2019)	not found

^{1}H and ^{13}C NMR spectra of the final compounds 2b to 9b.

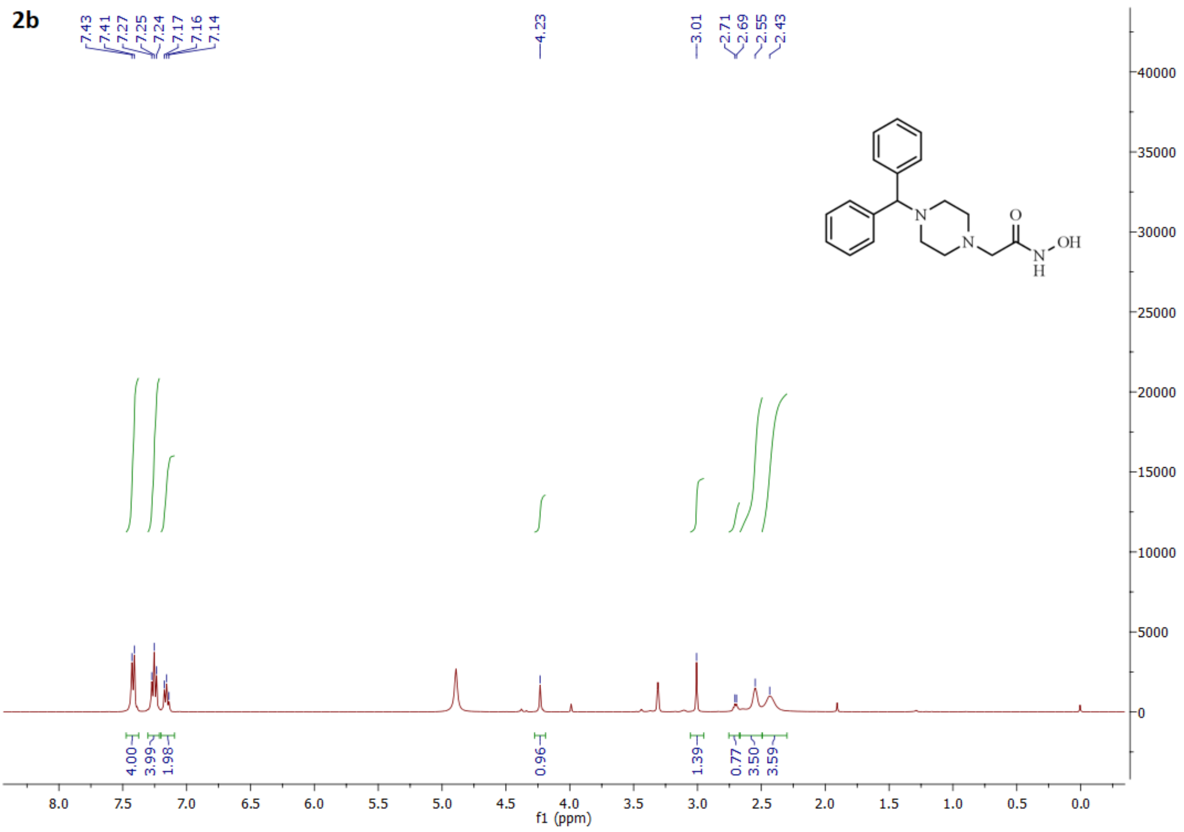

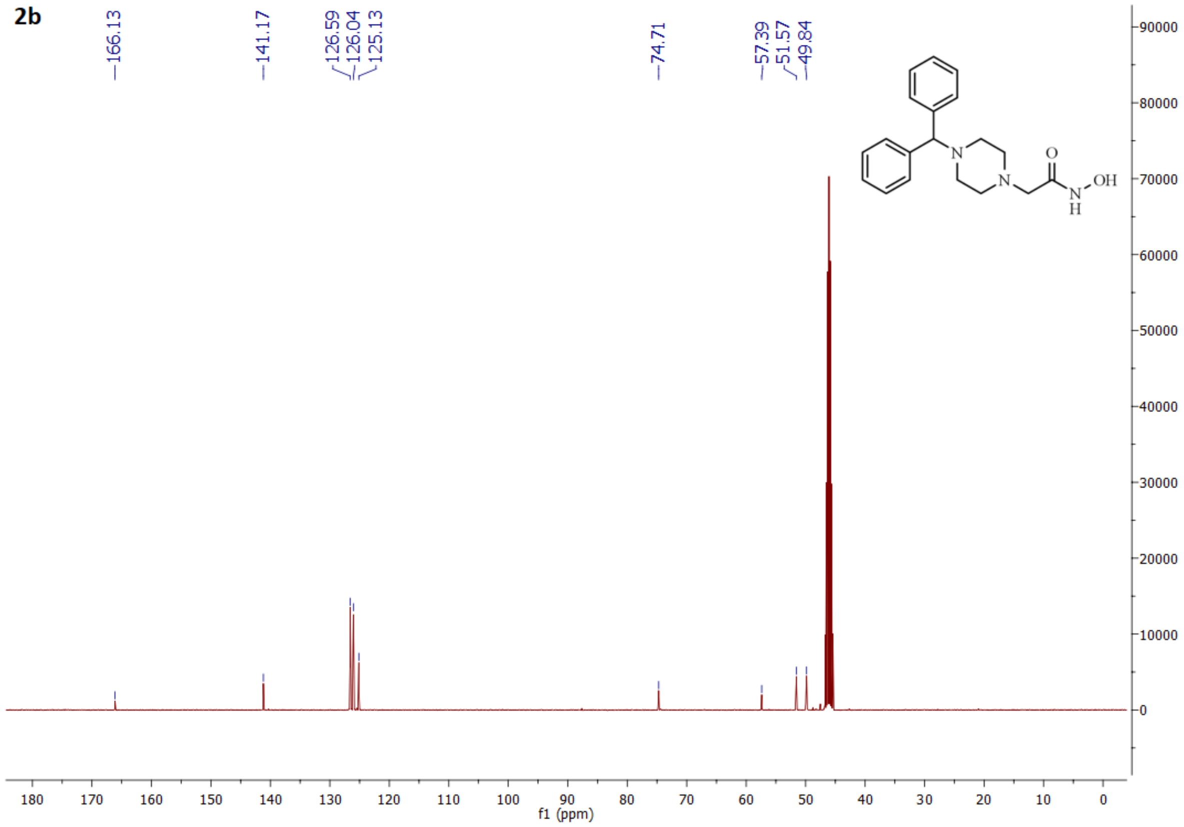

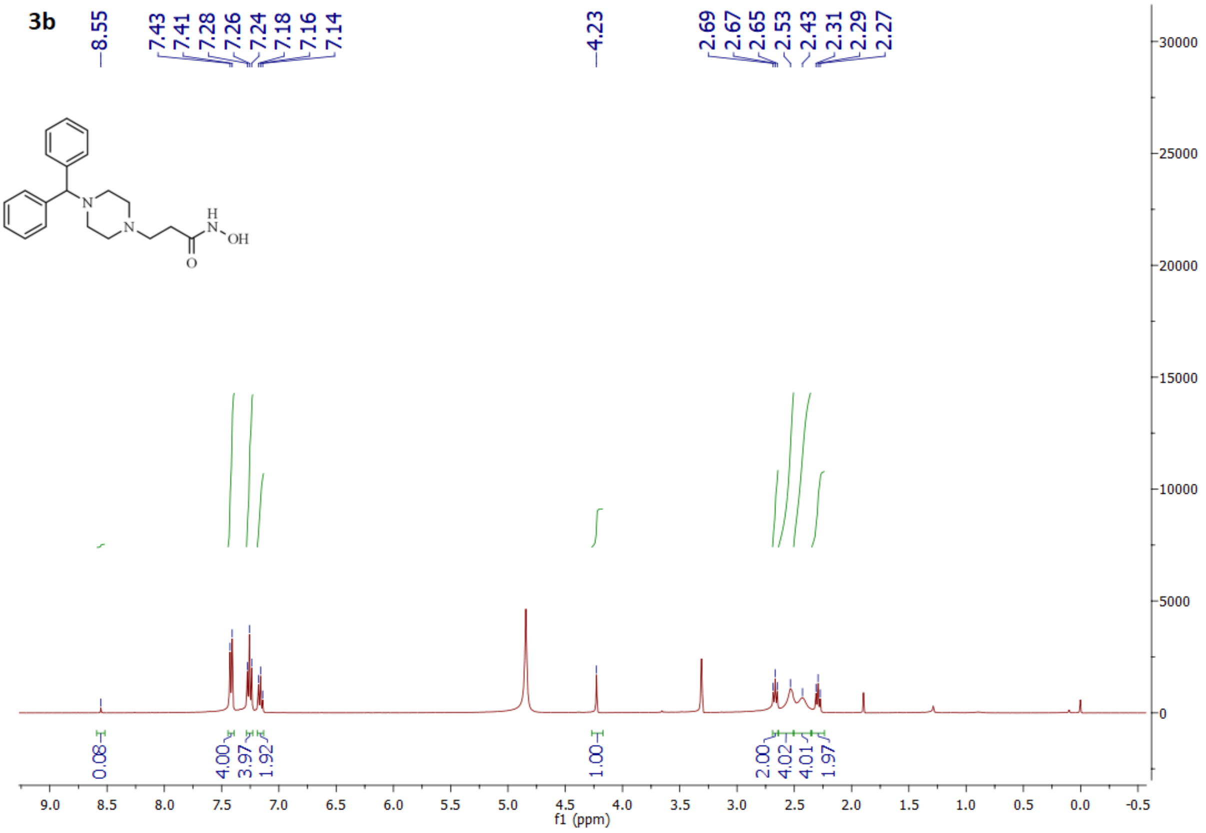
3b
8.55
7.43
7.41
7.28
7.26
7.24
7.18
7.16
7.14
4.23
2.69
2.67
2.65
2.53
2.43
2.31
2.29
2.27
0.08
4.00
3.97
1.92
1.00
2.00
4.02
4.01
1.97
f1 (ppm)

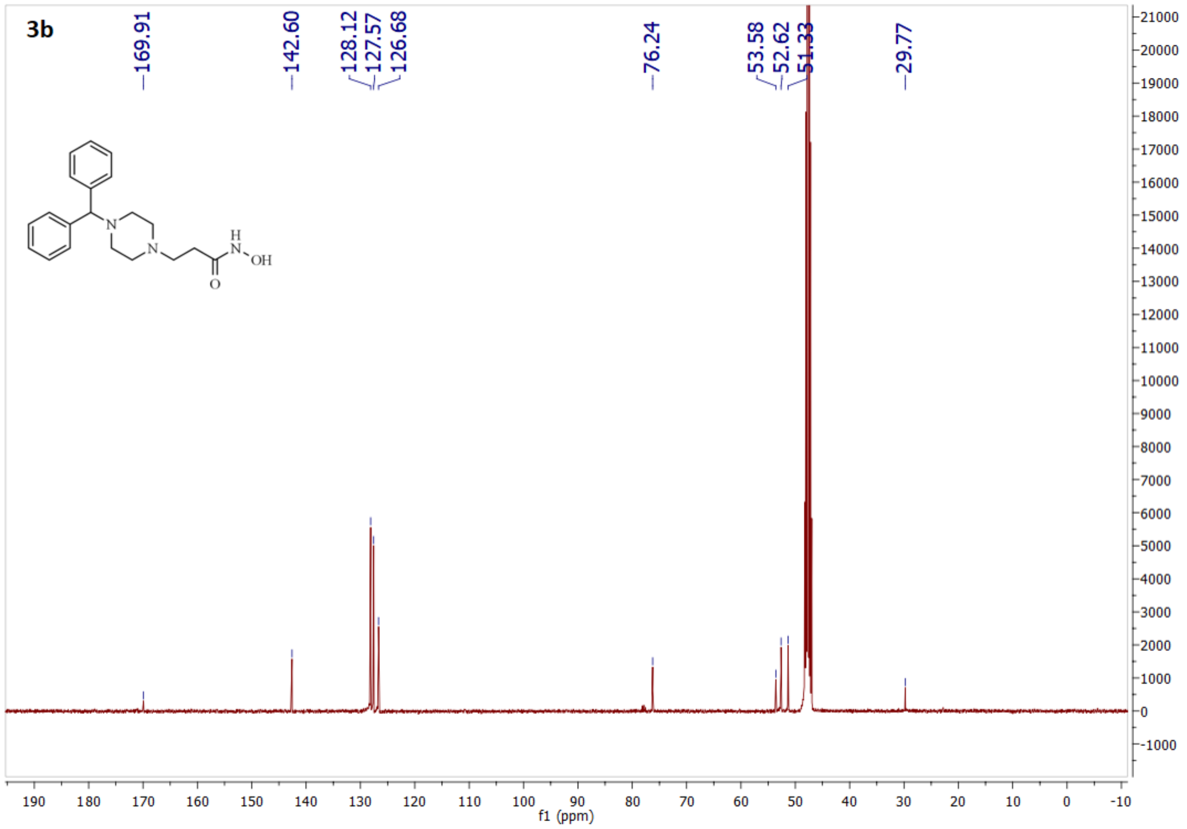
3b
169.91
142.60
128.12
127.57
126.68
76.24
53.58
52.62
51.33
29.77
f1 (ppm)

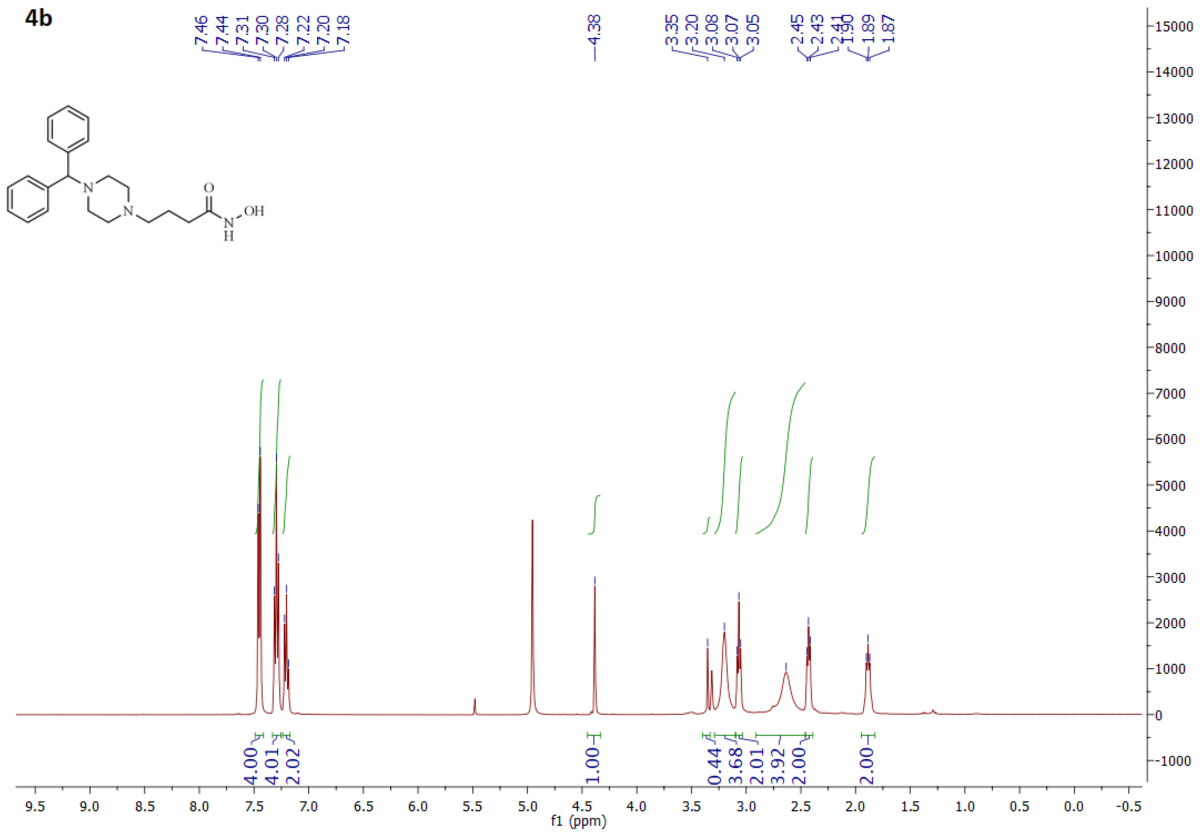
4b
7.46
7.44
7.31
7.30
7.28
7.22
7.20
7.18
4.38
3.35
3.20
3.08
3.07
3.05
2.45
2.43
2.41
1.90
1.89
1.87
4.00
4.01
2.02
1.00
0.44
3.68
2.01
3.92
2.00
2.00
f1 (ppm)

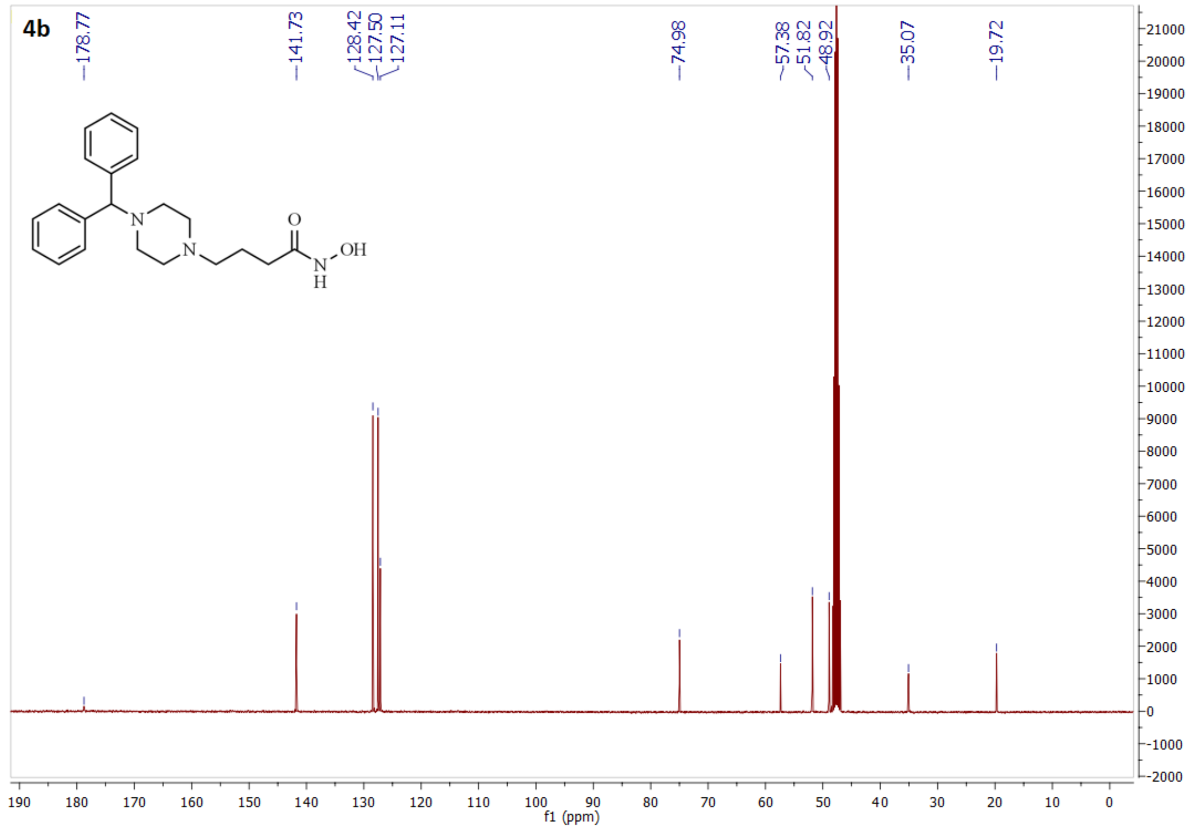
4b
178.77
141.73
128.42
127.50
127.11
74.98
57.38
51.82
48.92
35.07
19.72
f1 (ppm)

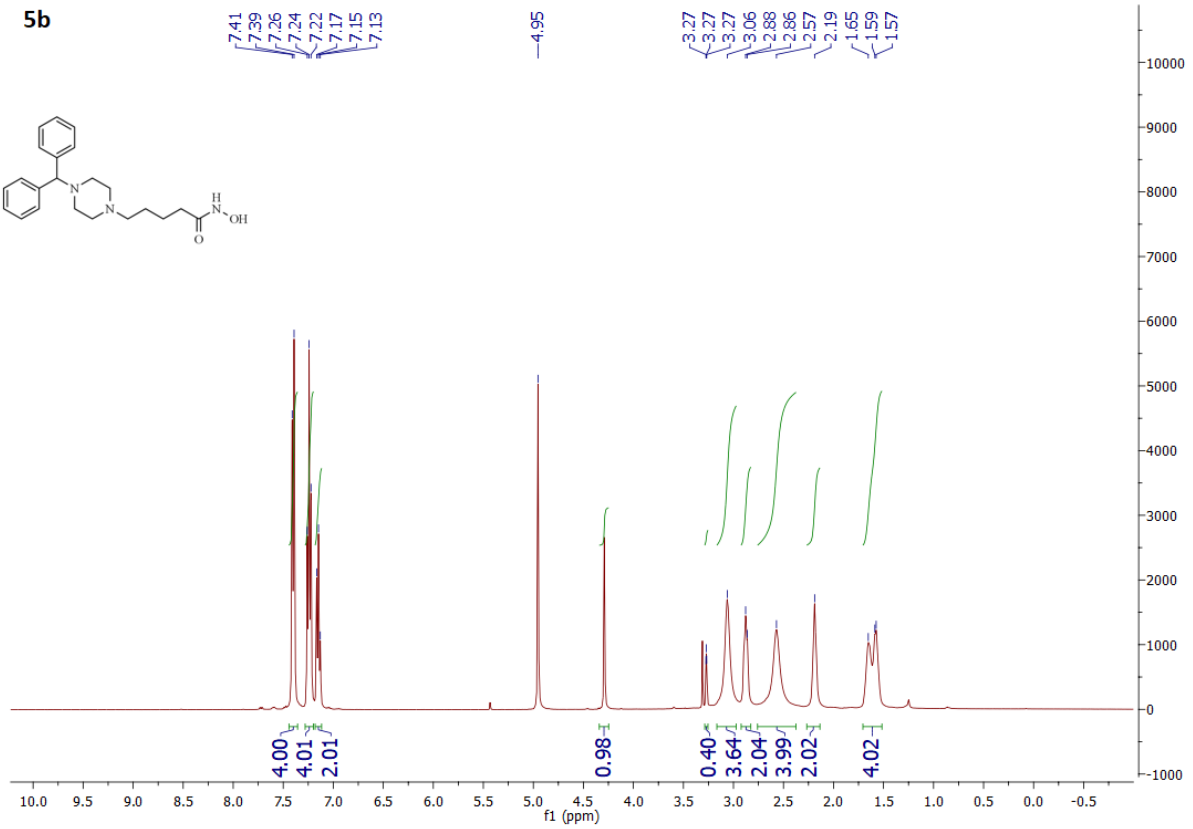

5b
7.41
7.39
7.26
7.24
7.22
7.17
7.15
7.13
4.95
3.27
3.27
3.27
3.06
2.88
2.86
2.57
2.19
1.65
1.59
1.57
4.00
4.01
2.01
0.98
0.40
3.64
2.04
3.99
2.02
4.02
f1 (ppm)

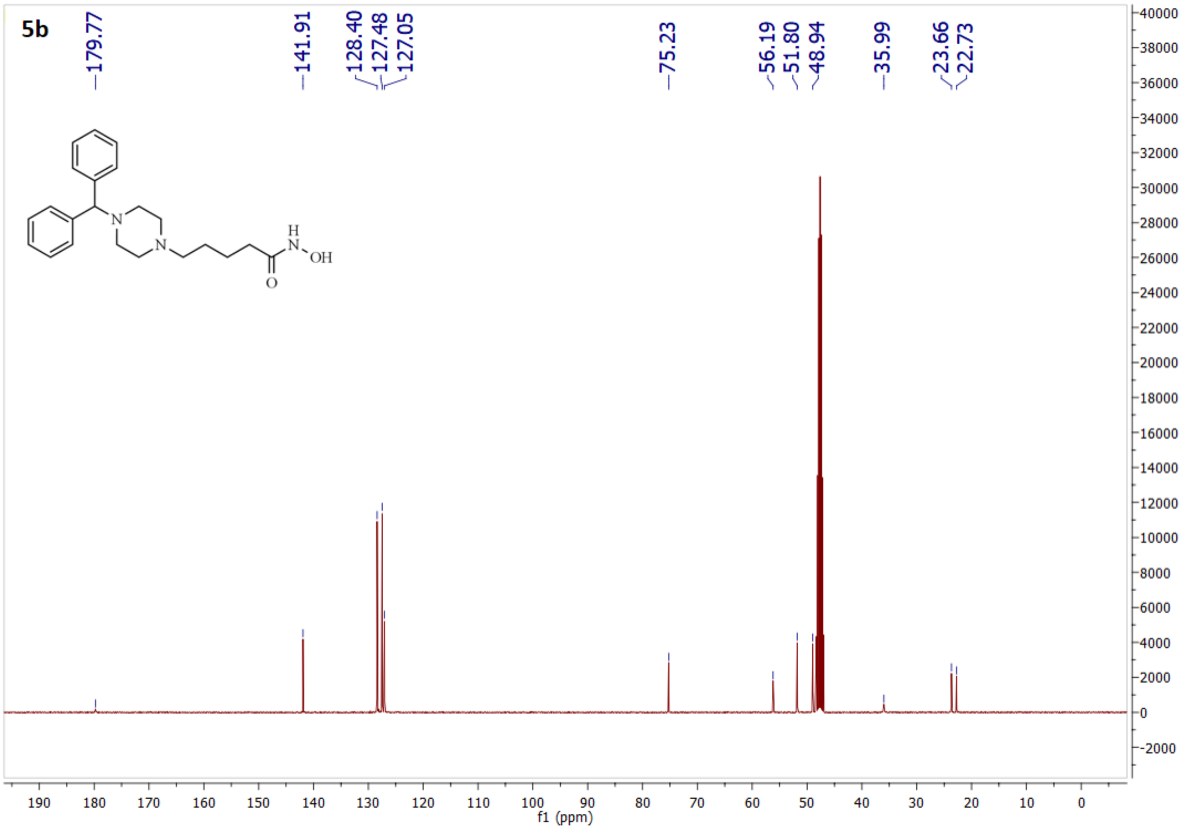

5b
179.77
141.91
128.40
127.48
127.05
75.23
56.19
51.80
48.94
35.99
23.66
22.73
f1 (ppm)

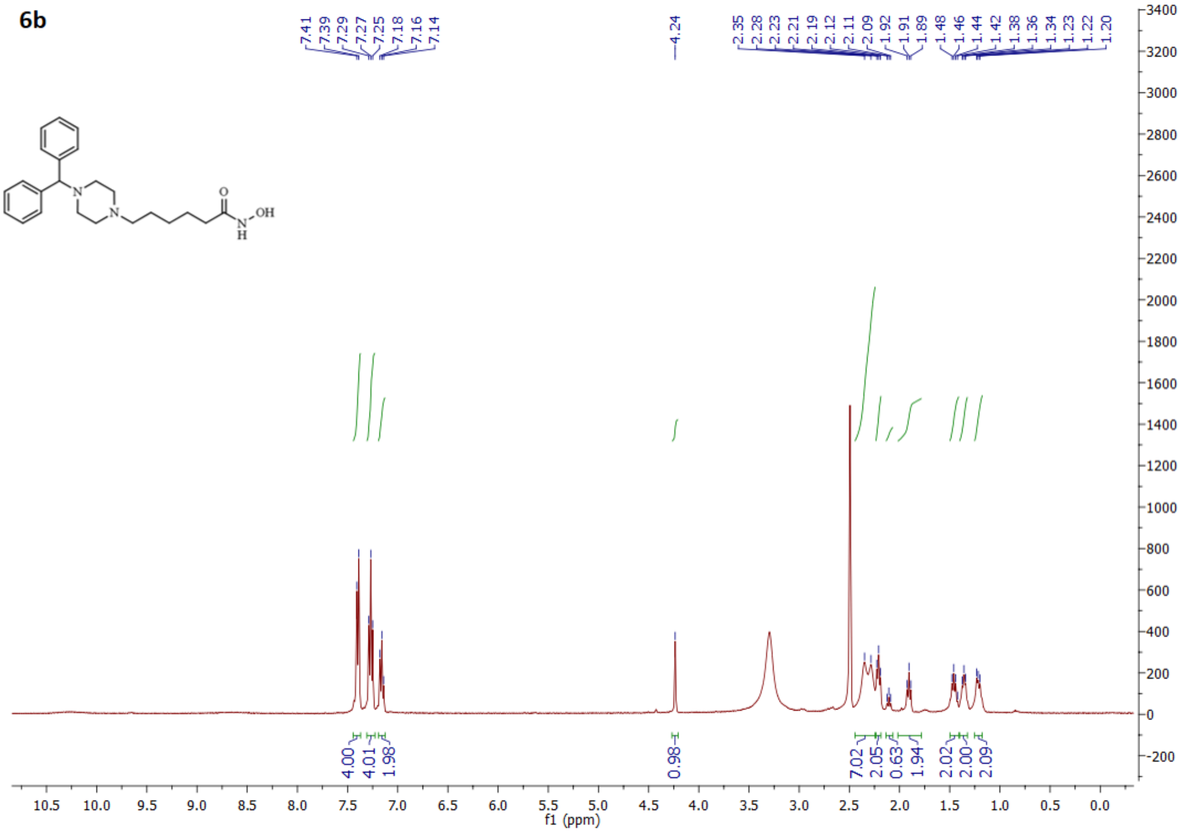
6b
7.41
7.39
7.29
7.27
7.25
7.18
7.16
7.14
4.24
2.35
2.28
2.23
2.21
2.19
2.12
2.11
2.09
1.92
1.91
1.89
1.48
1.46
1.44
1.42
1.38
1.36
1.34
1.23
1.22
1.20
4.00
4.01
1.98
0.98
7.02
2.05
0.63
1.94
2.02
2.00
2.09
f1 (ppm)

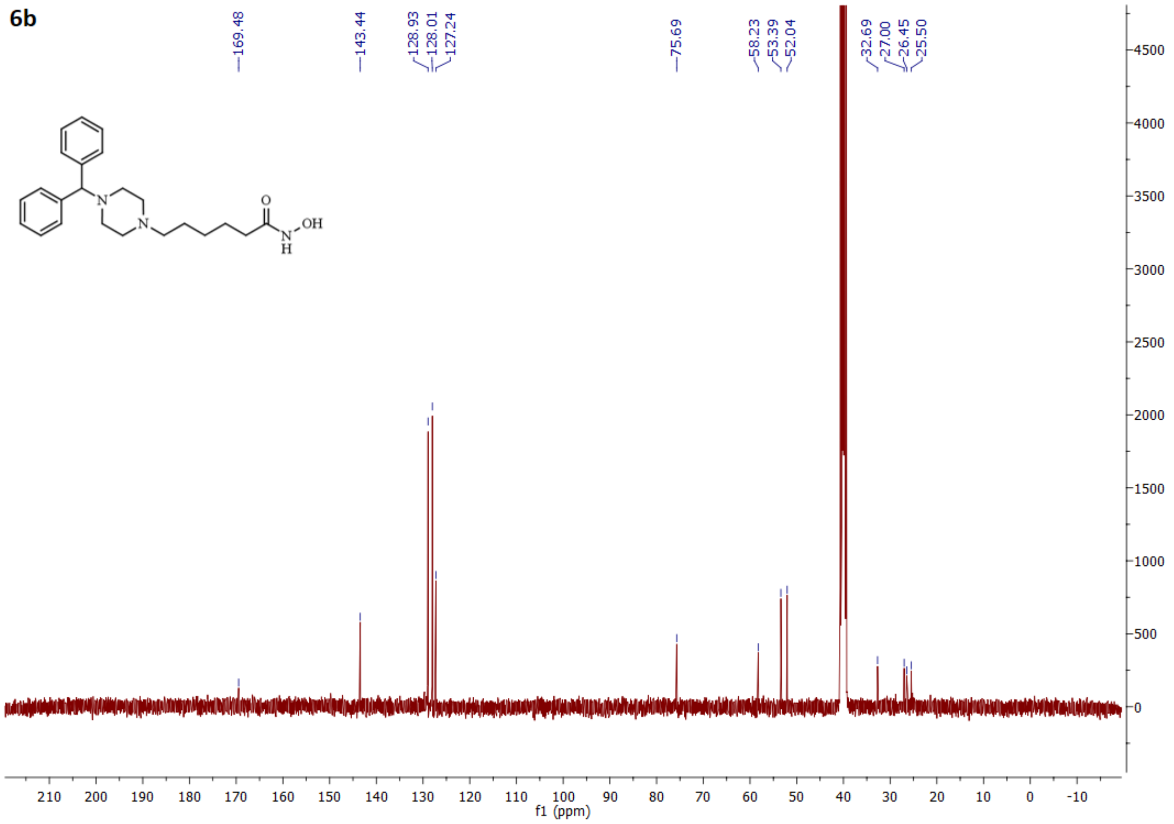
6b
169.48
143.44
128.93
128.01
127.24
75.69
58.23
53.39
52.04
32.69
27.00
26.45
25.50
f1 (ppm)

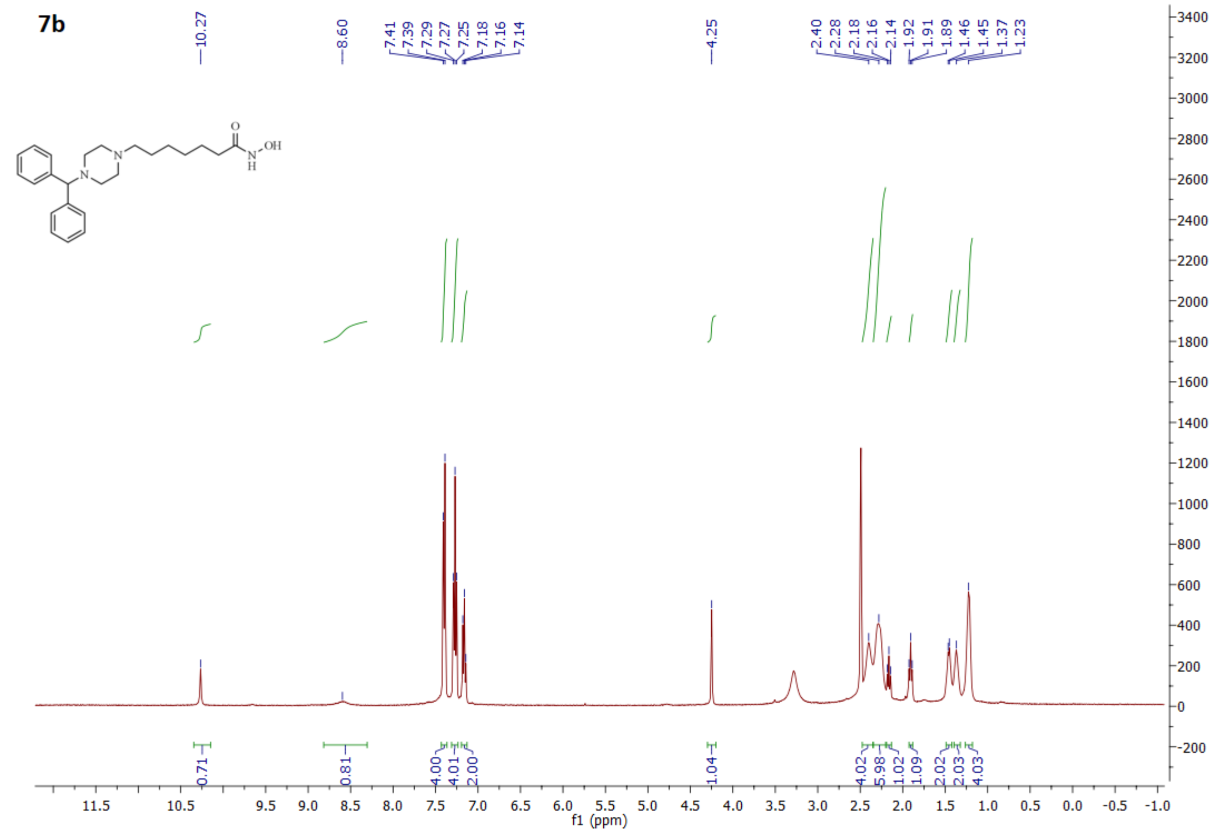
7b
10.27
8.60
7.41
7.39
7.29
7.27
7.25
7.18
7.16
7.14
4.25
2.40
2.28
2.18
2.16
2.14
1.92
1.91
1.89
1.46
1.45
1.37
1.23
0.71
0.81
4.00
4.01
2.00
1.04
4.02
5.98
1.02
1.09
2.02
2.03
4.03
f1 (ppm)

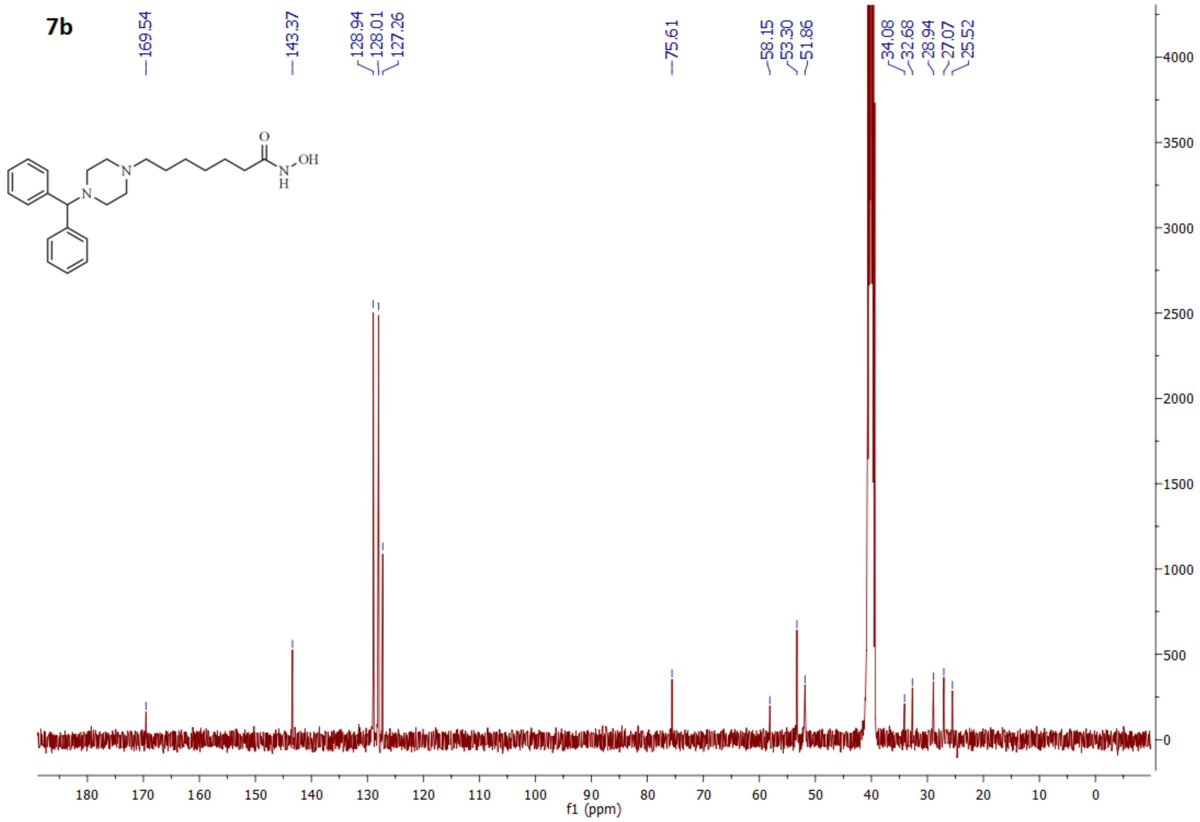
7b
169.54
143.37
128.94
128.01
127.26
75.61
58.15
53.30
51.86
34.08
32.68
28.94
27.07
25.52
f1 (ppm)

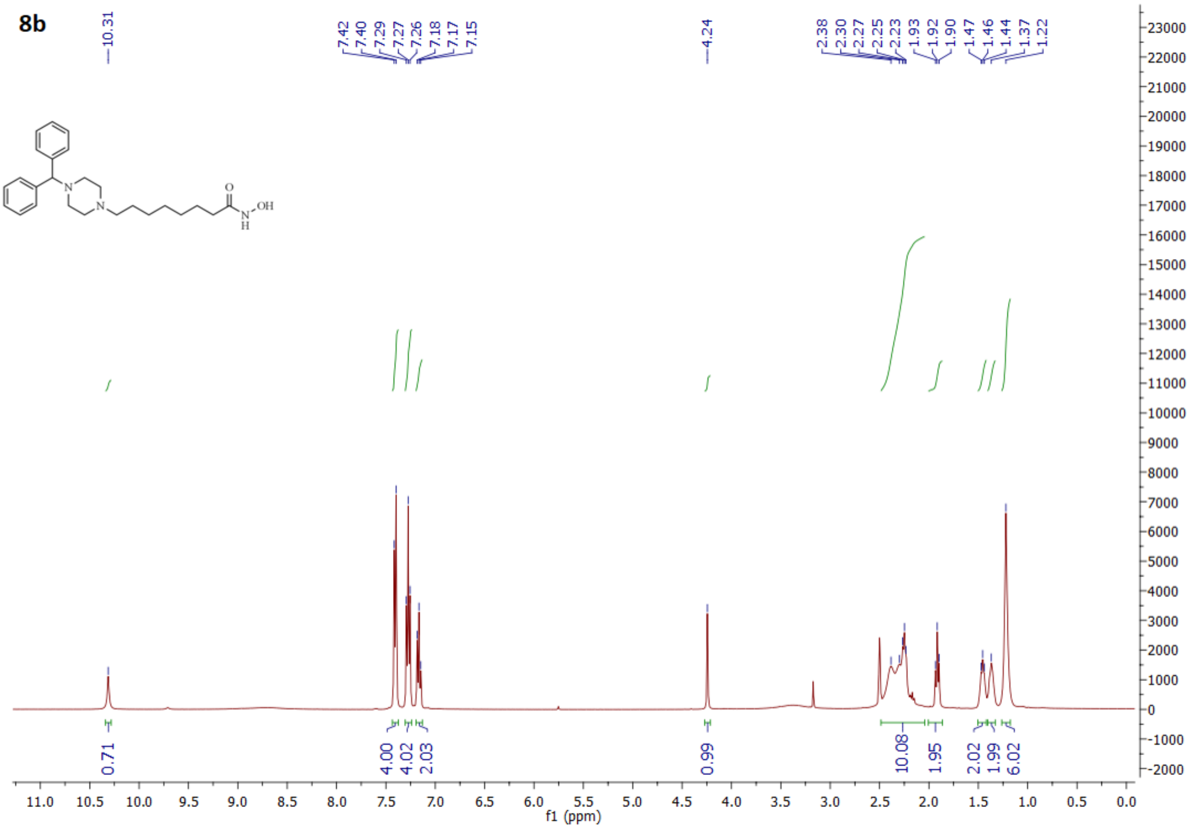
8b
10.31
7.42
7.40
7.29
7.27
7.26
7.18
7.17
7.15
4.24
2.38
2.30
2.27
2.25
2.23
1.93
1.92
1.90
1.47
1.46
1.44
1.37
1.22
0.71
4.00
4.02
2.03
0.99
10.08
1.95
2.02
1.99
6.02
f1 (ppm)

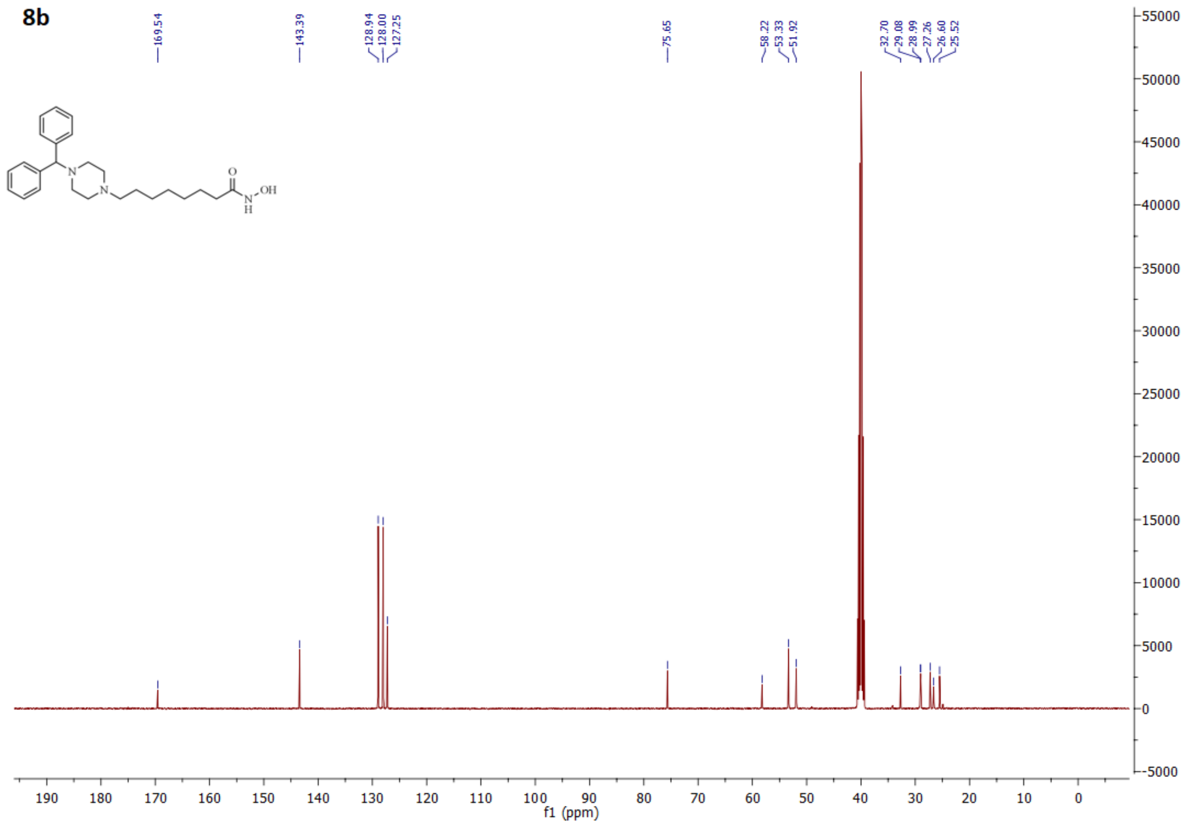
8b
169.54
143.39
128.94
128.00
127.25
75.65
58.22
53.33
51.92
32.70
29.08
28.99
27.26
26.60
25.52
f1 (ppm)

9b

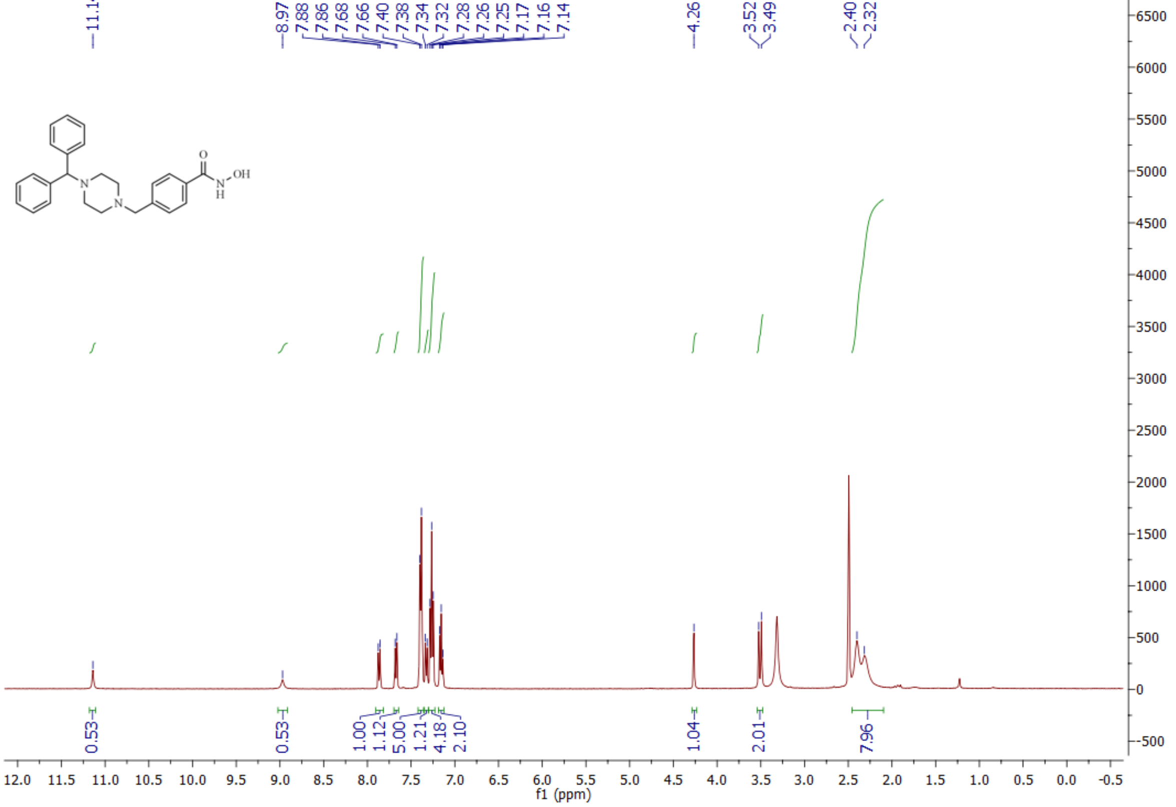
11.14
8.97
7.88
7.86
7.68
7.66
7.40
7.38
7.34
7.32
7.28
7.26
7.25
7.17
7.16
7.14
4.26
3.52
3.49
2.40
2.32
0.53
0.53
1.00
1.12
5.00
1.21
4.18
2.10
1.04
2.01
7.96
f1 (ppm)

9b

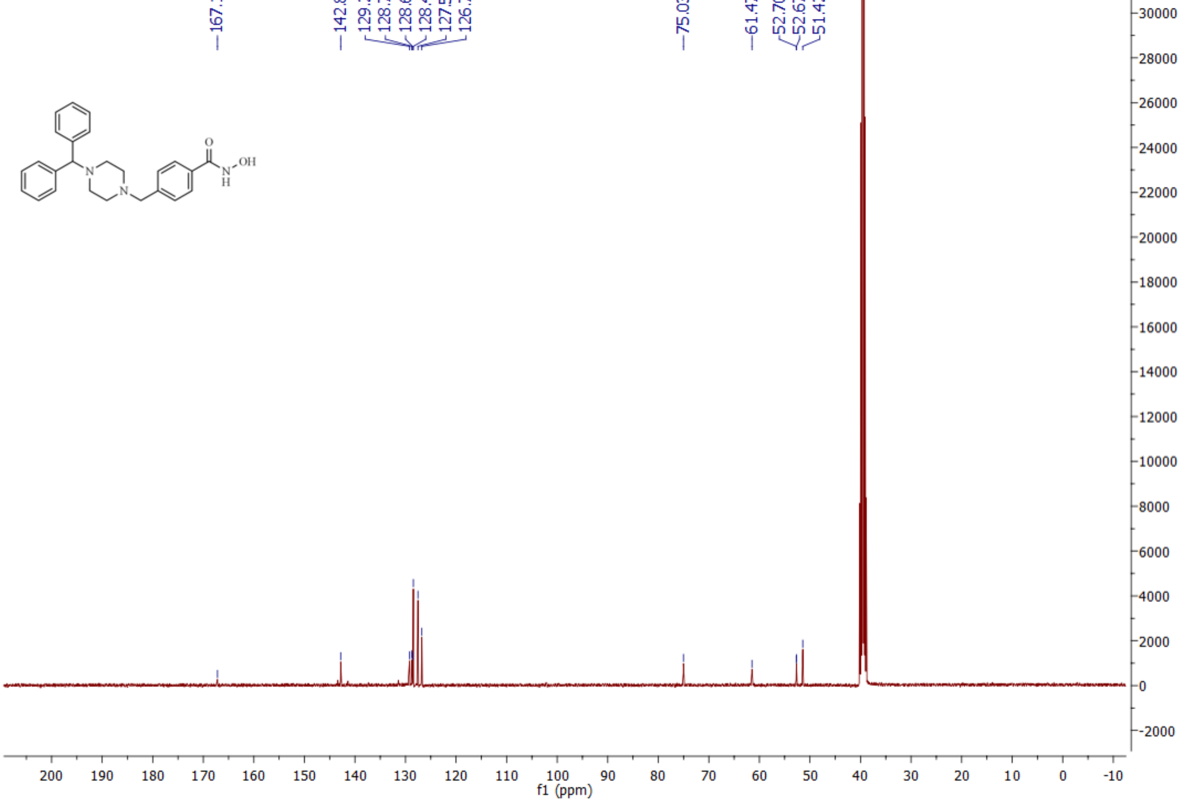
167.19
142.83
129.20
128.74
128.63
128.45
127.54
126.78
75.03
61.47
52.70
52.67
51.42
f1 (ppm)

LC-ESI (+) HRMS (ToF) mass spectra for compounds 6b – 9b in a range (m/z 100–1000)

Compound **6b**

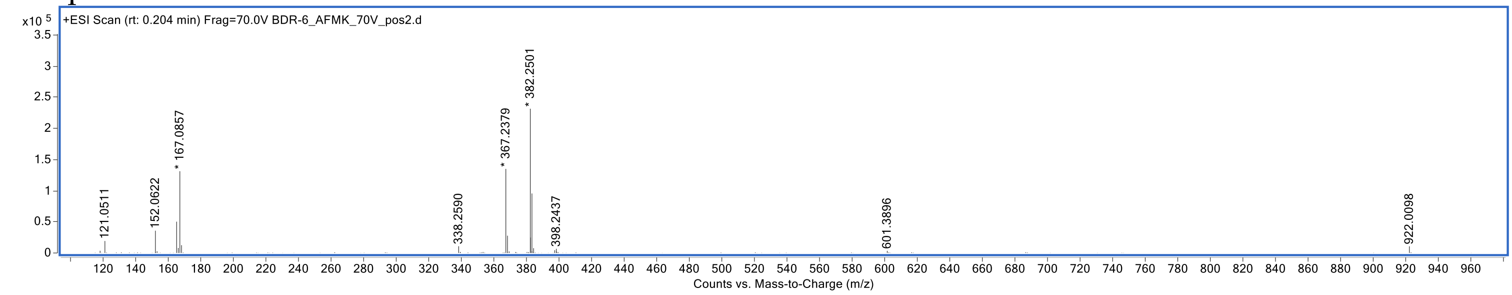

Compound **7b**

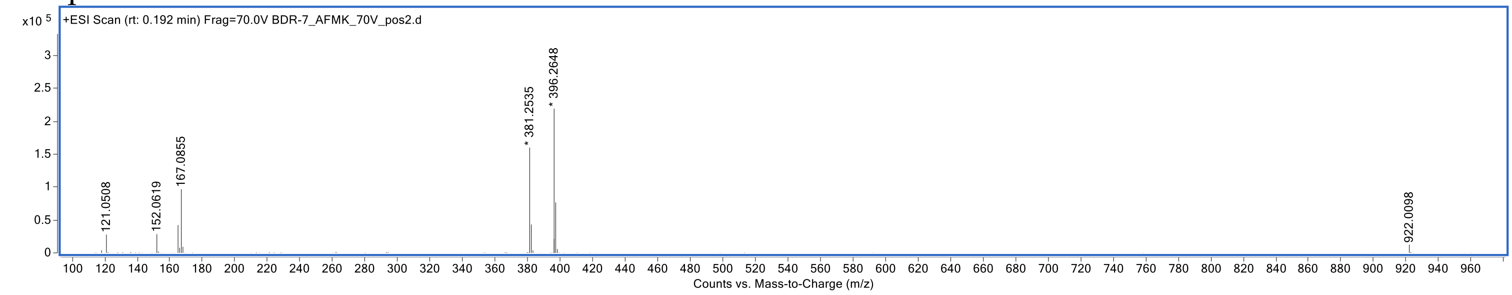

Compound **8b**

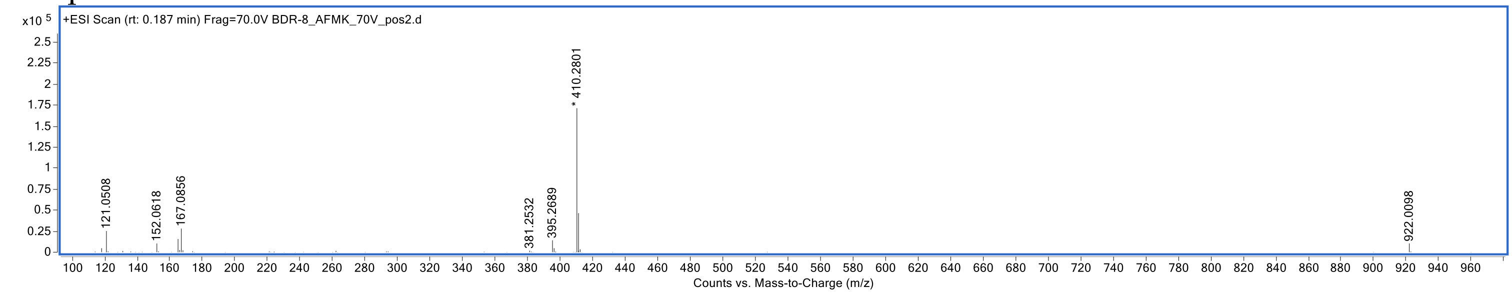

Compound **9b**

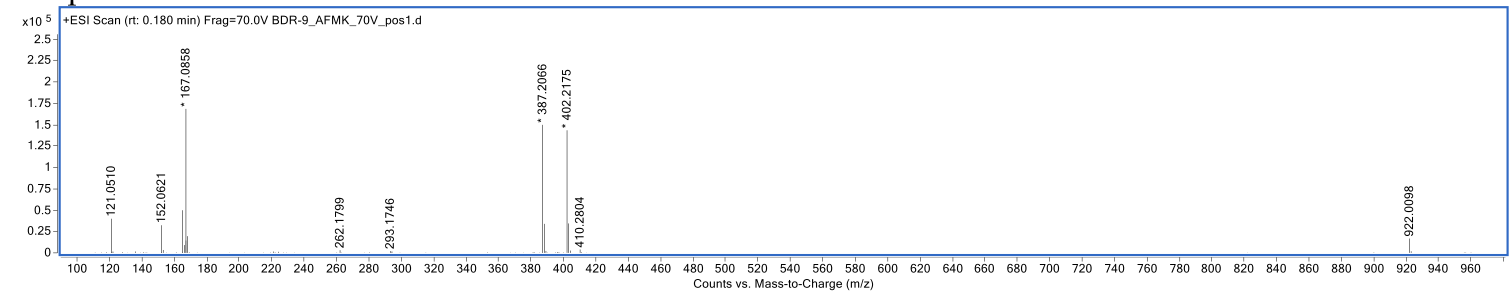

Note S1. Molecular dynamics simulations

Molecular dynamics simulations were performed using Groningen Machine for Chemical Simulation (GROMACS) package (version 2020.4)(Abraham et al., 2015). Initial coordinates of HDAC6:inhibitor complexes were obtained through molecular docking. Crystal waters and potassium ions were retained from PDB: 5EDU. Protein and ligand force field parameters were assigned using the Amber ff99SB-ILDN force field (Lindorff-Larsen et al., 2010) and General Amber force field (GAFF2) (Vassetti et al., 2019), respectively. The partial atomic charges of ligand were assigned using Restrained Electrostatic Potential (RESP) method at HF-6-31G* level of theory. Topologies and parameters for ligands were assigned and converted in GROMACS format using the AnteChamber Python Parser interfacE (ACPYPE) (Sousa da Silva & Vranken, 2012). The protein-ligand complexes were solvated with TIP3P water model, and neutralized with Na^+ counterions in octahedral periodic box. Systems were minimized with steepest descent algorithm with maximum force set to set to 10 kJ/mol nm^{-1} and 5000 steps. Subsequent equilibration of the-systems was performed firstly in NVT ensamble for 500 ps at 310 K using V-rescale thermostat. Secondly, equilibration in NPT ensemble was performed maintaining the pressure at 1.0 bar with Parrinello-Rahman barostat. All atom position restrains were applied on protein atoms. Position restraints were gradually removed using force constants of 1000, 100 and 10 kJ/mol nm^{-2}. Each NPT equilibration step was performed for 500 ps of simulation. Production runs of 100 ns were performed for each system, with 2 fs time-step at constant temperature (310 K) and pressure (1 bar) using Particle Mesh Ewald (PME) approach for calculation of long-range electrostatic with cut-off values of 10 Å for non-bonded interactions. LINCS algorithm was used to constrain all bonds with H-atoms. RMSD and distance analysis of trajectories were performed using analysis tools from VMD 1.9.4 (Humphrey et al., 1996). Representative structures of HDAC6:inhibitor complexes had been obtained after Gromos clustering of trajectories using built-in Gromacs tool gmx cluster (Daura et al., 1999). Molecular graphics were generated using PyMOL 2.5 (Schrodinger, LLC. 2010. The PyMOL Molecular Graphics System, Version 2.5. https://github.com/schrodinger/pymol-open-source).

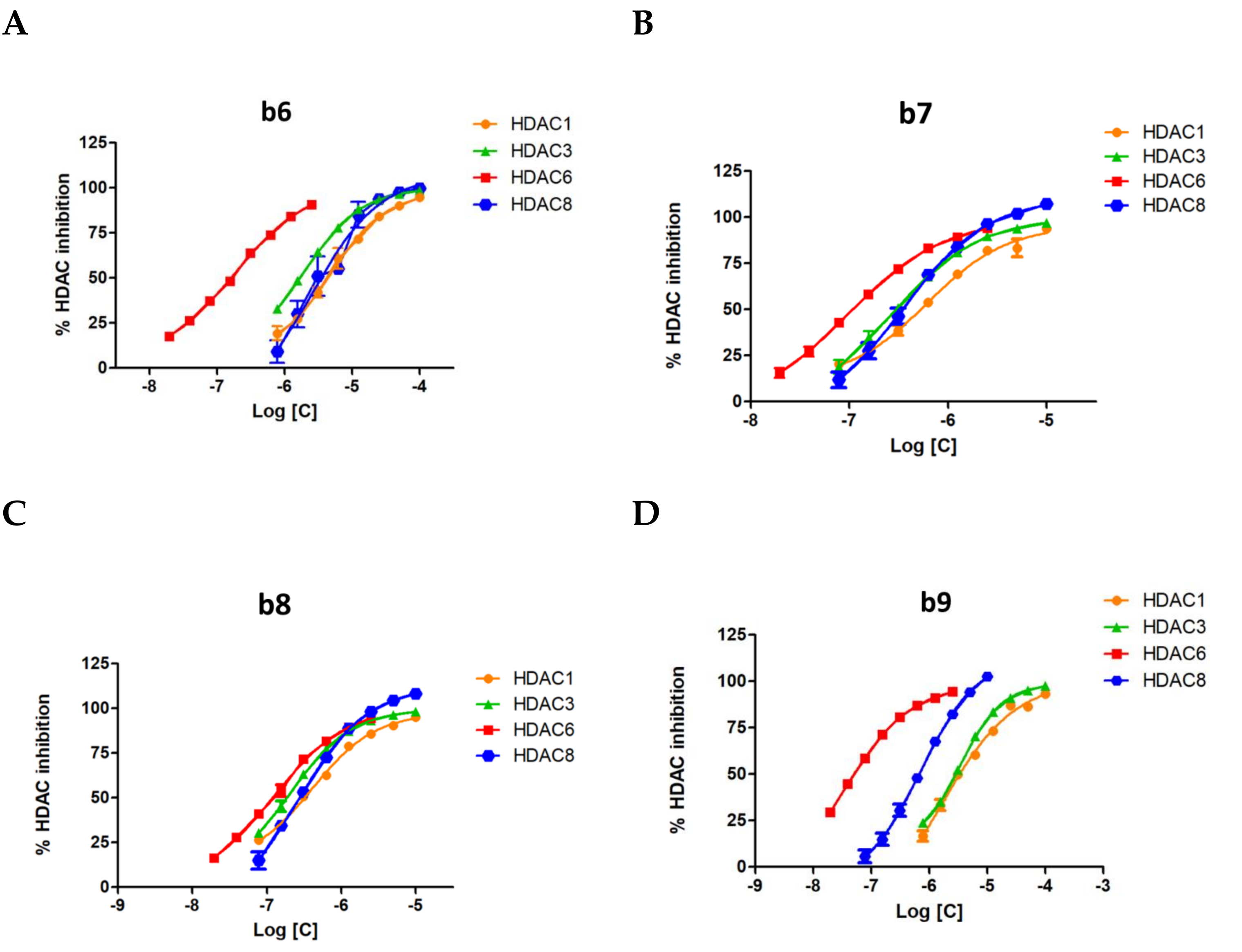

Figure S1. HDAC inhibition profiles of **6b** (**A**), **7b** (**B**), **8b** (**C**) and **9b** (**D**).

A

B

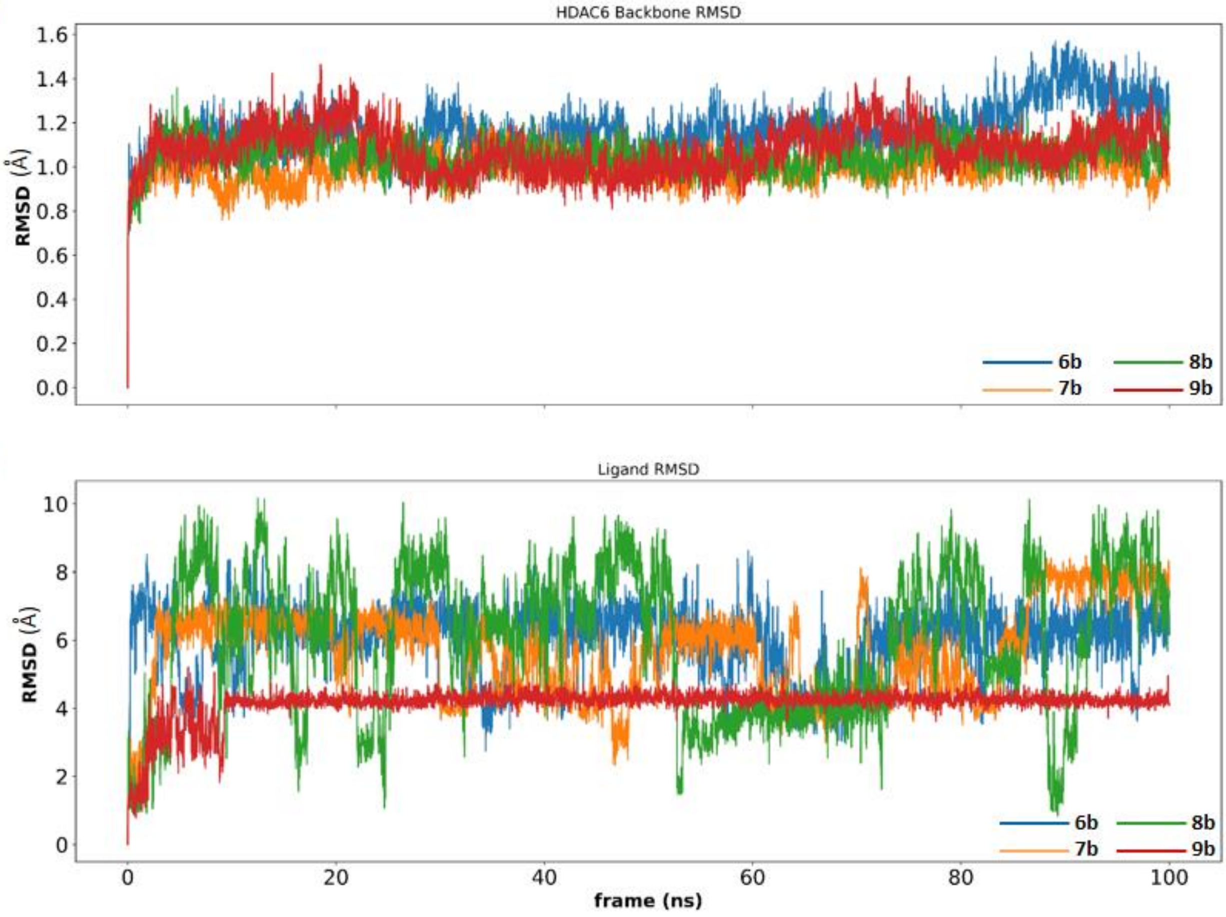

Figure S2. Root-Mean-Square-Deviation (RMSD) analysis of obtained trajectories.
(**A**) RMSD plot of HDAC6 backbone atoms fluctuations observed during 100 ns of MD simulations.
(**B**) RMSD plot of ligand atom fluctuations observed during 100 ns of MD simulations.

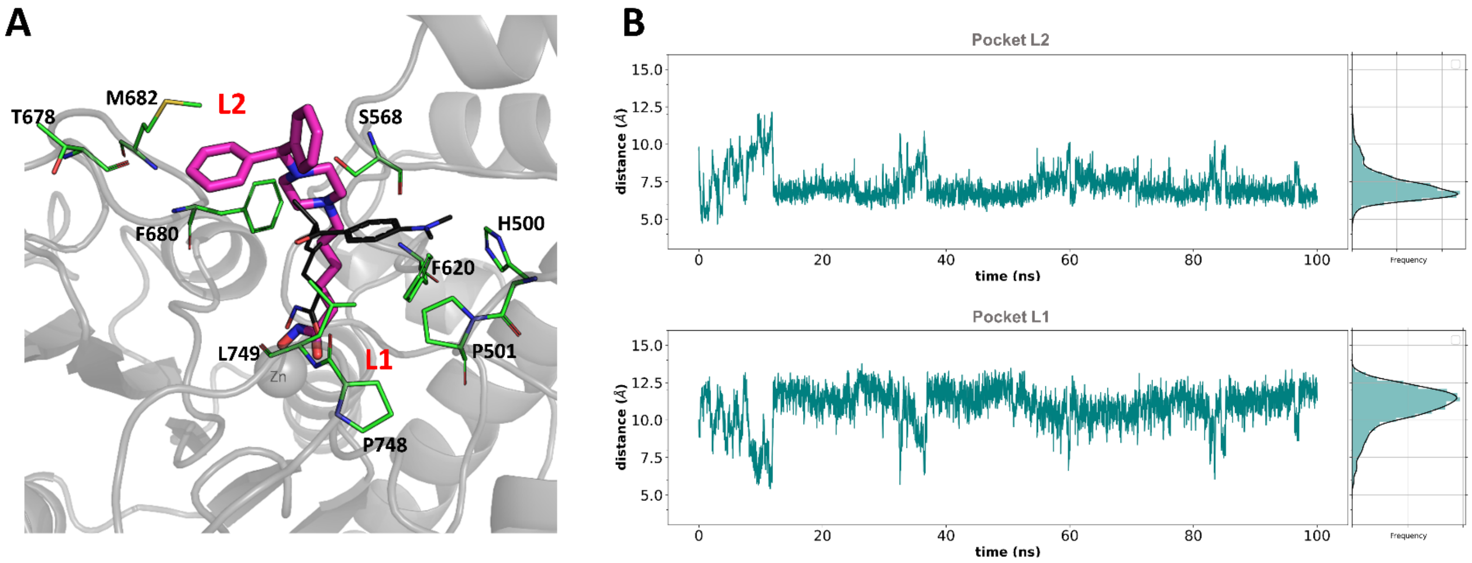

Figure S3. Predicted binding mode of **6b** and interaction of CAP group with L2 pocket of HDAC6. (**A**) Representation of the most populated binding mode of **6b** (magenta sticks) in complex with HDAC6 (gray cartoon) obtained after clustering of MD trajectory. Thin green sticks represent residues of the HDAC6 L1 and L2 binding pockets. **TSA** ligand (black lines) from PDB: 5EDU was used for pairwise comparison of binding modes. Residues are labeled with black, while pockets L1 and L2 are labeled with red letters. (**B**) Distances between centers of masses of CAP group and L2 pocket (upper plot) or L1 pocket (lower plot) observed during 100 ns of MD simulations.

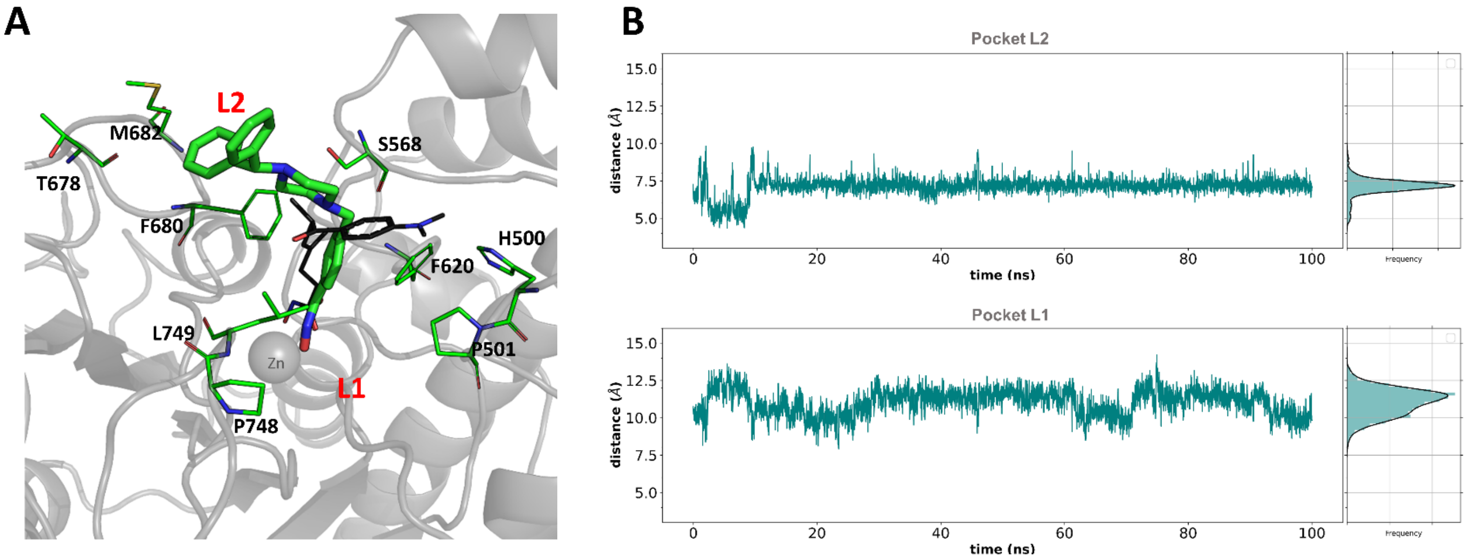

Figure S4. Predicted binding mode of **9b** and interaction of CAP group with L2 pocket of HDAC6.
(**A**) Representation of the most populated binding mode of **9b** (green sticks) in complex with HDAC6 (gray cartoon) obtained after clustering of MD trajectory. Thin green sticks represent residues of the HDAC6 L1 and L2 binding pockets. **TSA** ligand (black lines) from PDB: 5EDU was used for pairwise comparison of binding modes. Residues are labeled with black, while pockets L1 and L2 are labeled with red letters. (**B**) Distances between centers of masses of CAP group and L2 pocket (upper plot) or L1 pocket (lower plot) observed during 100 ns of MD simulations.

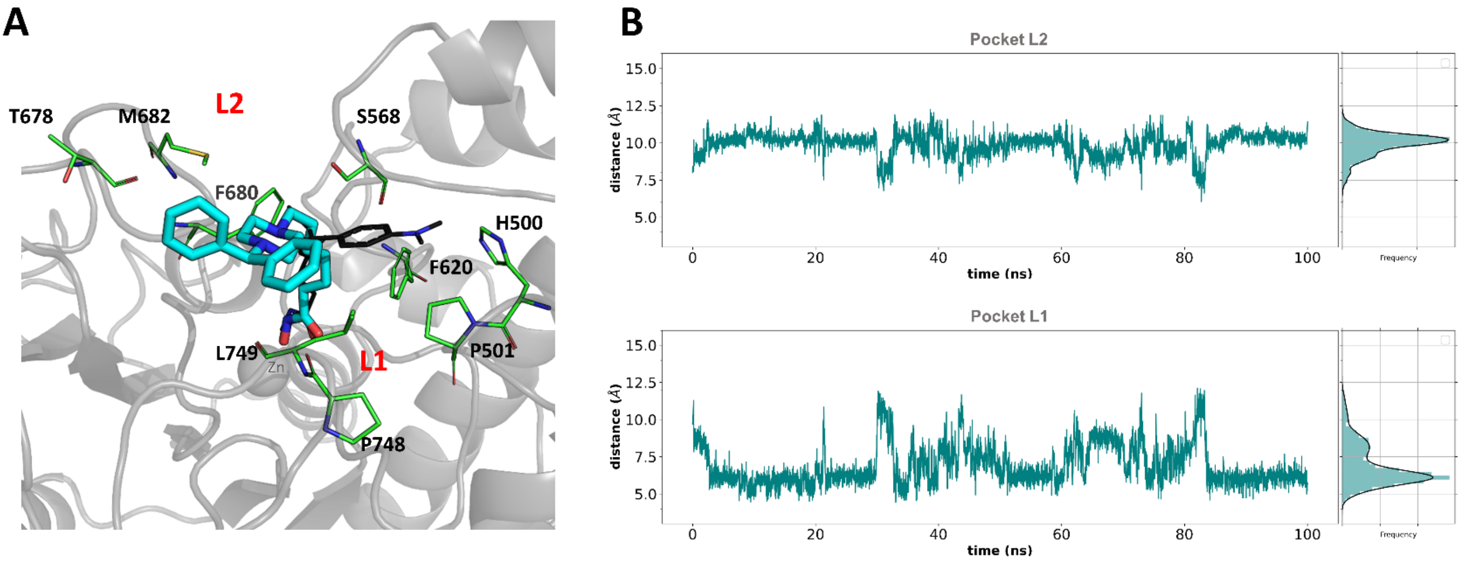

Figure S5. Predicted binding mode of **7b** and interaction of CAP group with L1 pocket of HDAC6. (**A**) Representation of the most populated binding mode of **7b** (cyan sticks) in complex with HDAC6 (gray cartoon) obtained after clustering of MD trajectory. Thin green sticks represent residues of the HDAC6 L1 and L2 binding pockets. **TSA** ligand (black lines) from PDB: 5EDU was used for pairwise comparison of binding modes. Residues are labeled with black, while pockets L1 and L2 are labeled with red letters. (**B**) Distances between centers of masses of CAP group and L2 pocket (upper plot) or L1 pocket (lower plot) observed during 100 ns of MD simulations.

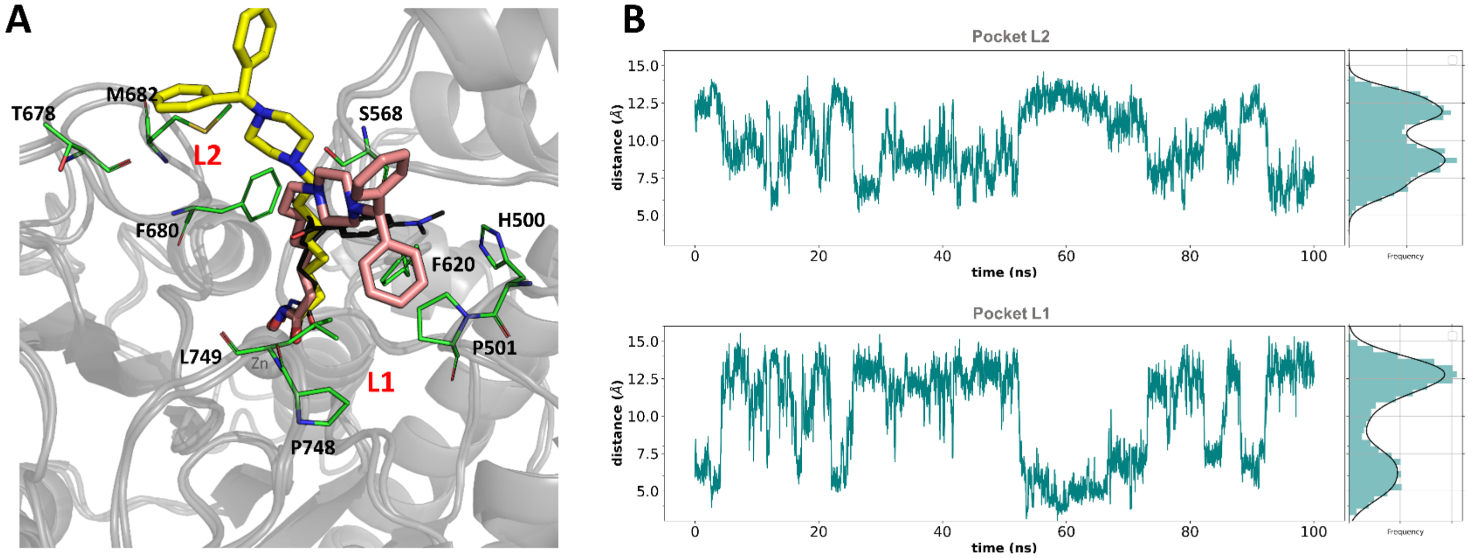

Figure S6. Predicted binding mode of **8b** and interaction of CAP group with L1 and L2 pockets of HDAC6. (**A**) Representation of the two the most populated binding modes of **8b** (yellow and salmon sticks) in complex with HDAC6 (gray cartoon) obtained after clustering of MD trajectory. Thin green sticks represent residues of the HDAC6 L1 and L2 binding pockets observed for one of the centroids (residues from other centroid are omitted for clarity). **TSA** ligand (black lines) from PDB: 5EDU was used for pairwise comparison of binding modes. Residues are labeled with black, while pockets L1 and L2 are labeled with red letters. (**B**) Distances between centers of masses of CAP group and L2 pocket (upper plot) or L1 pocket (lower plot) observed during 100 ns of MD simulations.

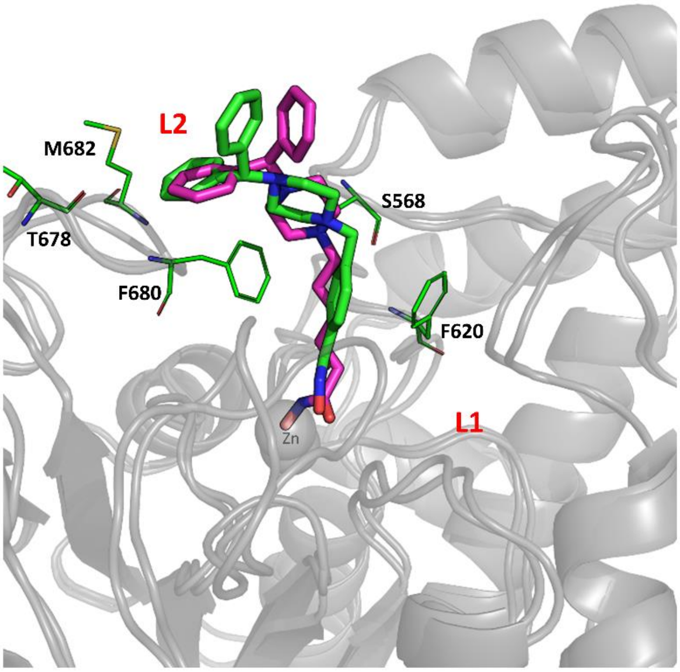

Figure S7. Pairwise comparison of predicted binding modes of **6b** (green sticks) and **9b** (magenta sticks) in interaction with HDAC6 (gray cartoon).
Residues from L1 pocket are presented in thin green sticks. Residues are labeled with black, while L1 and L2 pockets are labeled with red letters.

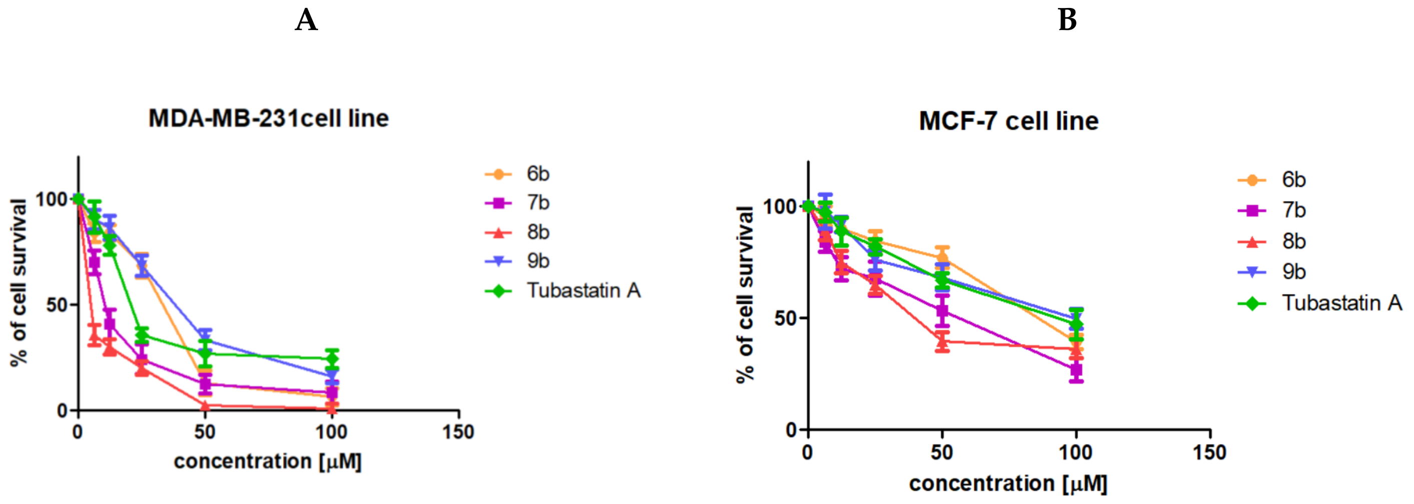

Figure S8. Cell viability data generated after treating MDA-MB-231 cells (**A**) and MCF-7 cells (**B**) for 48 h with synthesized inhibitors.

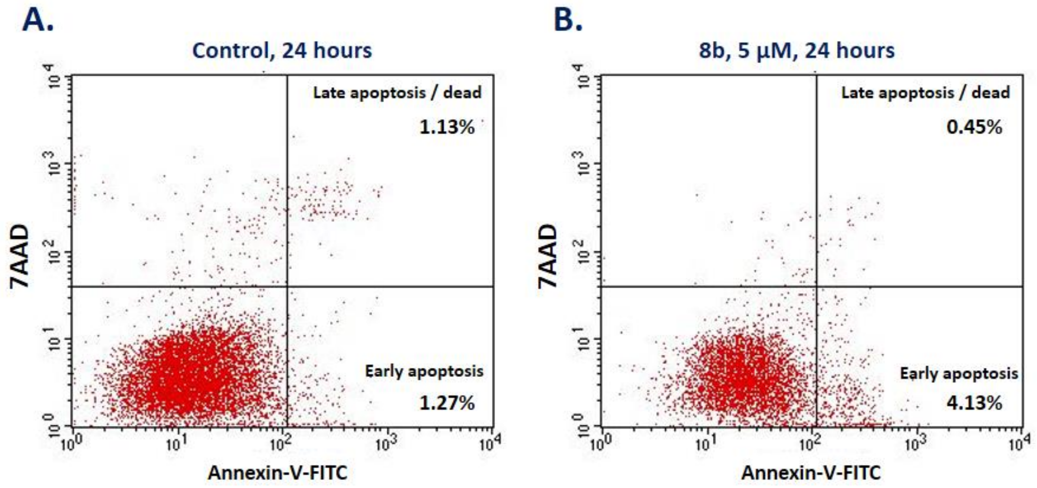

Figure S9. Flow cytometry Annexin V-FITC/7-AAD staining of early and late apoptotic cell population at 5 µM **8b** treatment of MDA-MB-231 cells. Population of early (4.13%) and late (0.45%) apoptotic cells after 5 µM **8b** treatment was not significantly higher than observed in control (1.27% early apoptotic population (**A**); 1.13% late apoptotic population (**B**)).

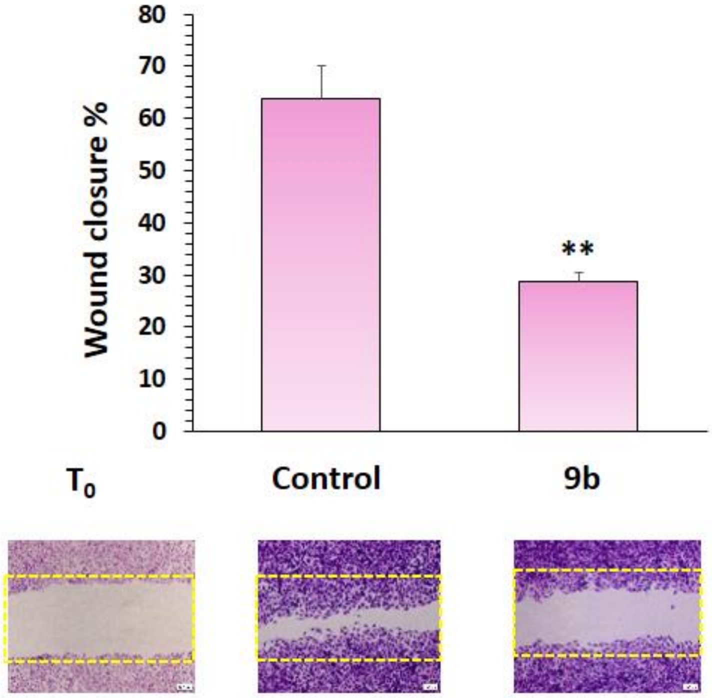

Figure S10. The HDAC6 inhibitor **9b** reduces MCF-7 cell migration.
Confluent MCF-7 monolayers were subjected to wound healing assay to determine HDAC6 inhibition on cell migration. After a scratch, cells were treated with 10 μM of **9b** for 24 h, fixed, and stained. **9b** significantly inhibits the closure of the wound compared with control cells. T_0, cells were fixed just after made the monolayer scratch. Magnification 40×, bar = 50 μm. Representative results from three independent experiments are shown. Significant difference between treatments by t-test: ** $p < 0.01$.

Table S2. Lethal and teratogenic effects observed in zebrafish (*Danio rerio*) embryos at different hours post fertilization (hpf).

Category	Toxicological parameters	Exposure time (hpf)				
		24	48	72	96	120
**Lethal effect**	Coagulated eggs[a]	•	•	•	•	•
	Lack of the heart beating	•	•	•	•	•
	Non-detachment of the tail	•	•	•	•	•
	Lack of somite formation	•	•	•	•	•
**Teratogenic effect**	Malformation of head	•	•	•	•	•
	Malformation of eyes[b]	•	•	•	•	•
	Malformation of sacculi/otoliths[c]	•	•	•	•	•
	Malformation of chorda	•	•	•	•	•
	Malformation of tail[d]	•	•	•	•	•
	Scoliosis	•	•	•	•	•
	Yolk edema	•	•	•	•	•
	Yolk deformation	•	•	•	•	•
	Growth retardation[e]		•	•	•	•
	Hatching			•	•	•
	Swimmbladder development					•
**Hepatotoxicity**	Yolk absorption			•	•	•
	Liver darkening			•	•	•
**Cardiotoxicity**	Pericardial edema		•	•	•	•
	Heart morphology			•	•	•
	Heart beating rate (beat/min)				•	•
**Melanocytotoxicity**	Skin pigmentation (melanization)[f]		•	•	•	•
	Melanocytes morphology[g]		•	•	•	•

[a] No clear organs structure is recognized.

[b] Malformation of eyes was recorded for the retardation in eye development and abnormality in shape and size.

[c] Presence of none, one or more than two otoliths per sacculus, as well as reduction and enlargement of otoliths and/or sacculi (otic vesicles).

[d] Tail malformation was recorded when the tail was bent, twisted or shorter than to control embryos as assessed by optical comparation.

[e] Growth retardation was recorded by comparing with the control embryos in a body length (after hatching, at and onwards 72 hpf) using by optical comparison using an inverted microscope (CKX41; Olympus, Tokyo, Japan).

[f] Skin depigmentation of hyperpigmentation was assessed by optical comparation.

[g] Change in stellate morphology of the skin melanocytes was visually recorded

References

Abraham, M. J., Murtola, T., Schulz, R., Páll, S., Smith, J. C., Hess, B., & Lindahl, E. (2015). GROMACS: High performance molecular simulations through multi-level parallelism from laptops to supercomputers. *SoftwareX*, *1–2*, 19–25. https://doi.org/10.1016/j.softx.2015.06.001

An, P., Chen, F., Li, Z., Ling, Y., Peng, Y., Zhang, H., Li, J., Chen, Z., & Wang, H. (2020). HDAC8 promotes the dissemination of breast cancer cells via AKT/GSK-3β/Snail signals. *Oncogene*, *39*(26), 4956–4969. https://doi.org/10.1038/s41388-020-1337-x

An, P., Li, J., Lu, L., Wu, Y., Ling, Y., Du, J., Chen, Z., & Wang, H. (2019). Histone deacetylase 8 triggers the migration of triple negative breast cancer cells via regulation of YAP signals. *European Journal of Pharmacology*, *845*, 16–23. https://doi.org/10.1016/j.ejphar.2018.12.030

Daura, X., Gademann, K., Jaun, B., Seebach, D., van Gunsteren, W. F., & Mark, A. E. (1999). Peptide Folding: When Simulation Meets Experiment. *Angewandte Chemie International Edition*, *38*(1–2), 236–240. https://doi.org/10.1002/(SICI)1521-3773(19990115)38:1/2<236::AID-ANIE236>3.0.CO;2-M

Duan, B., Ye, D., Zhu, S., Jia, W., Lu, C., Wang, G., Guo, X., Yu, Y., Wu, C., & Kang, J. (2017). HDAC10 promotes angiogenesis in endothelial cells through the PTPN22/ERK axis. *Oncotarget*, *8*(37), 61338–61349. https://doi.org/10.18632/oncotarget.18130

Hsieh, T.-H., Hsu, C.-Y., Tsai, C.-F., Long, C.-Y., Chai, C.-Y., Hou, M.-F., Lee, J.-N., Wu, D.-C., Wang, S.-C., & Tsai, E.-M. (2014). MiR-125a-5p is a prognostic biomarker that targets HDAC4 to suppress breast tumorigenesis. *Oncotarget*, *6*(1), 494–509. https://doi.org/10.18632/oncotarget.2674

Hsieh, T.-H., Hsu, C.-Y., Tsai, C.-F., Long, C.-Y., Wu, C.-H., Wu, D.-C., Lee, J.-N., Chang, W.-C., & Tsai, E.-M. (2015). HDAC Inhibitors Target HDAC5, Upregulate MicroRNA-125a-5p, and Induce Apoptosis in Breast Cancer Cells. *Molecular Therapy*, *23*(4), 656–666. https://doi.org/10.1038/mt.2014.247

Huang, Y., Jian, W., Zhao, J., & Wang, G. (2018). Overexpression of HDAC9 is associated with poor prognosis and tumor progression of breast cancer in Chinese females. *OncoTargets and Therapy*, *11*, 2177–2184. https://doi.org/10.2147/OTT.S164583

Humphrey, W., Dalke, A., & Schulten, K. (1996). VMD: Visual molecular dynamics. *Journal of Molecular Graphics*, *14*(1), 33–38. https://doi.org/10.1016/0263-7855(96)00018-5

Kaluza, D., Kroll, J., Gesierich, S., Yao, T.-P., Boon, R. A., Hergenreider, E., Tjwa, M., Rössig, L., Seto, E., Augustin, H. G., Zeiher, A. M., Dimmeler, S., & Urbich, C. (2011). Class IIb HDAC6 regulates endothelial cell migration and angiogenesis by deacetylation of cortactin. *The EMBO Journal*, *30*(20), 4142–4156. https://doi.org/10.1038/emboj.2011.298

Kashio, T., Shirakura, K., Kinoshita, M., Morita, M., Ishiba, R., Muraoka, K., Kanbara, T., Tanaka, M., Funatsu, R., Hino, N., Koyama, S., Suzuki, R., Yoshioka, Y., Aoshi, T., Doi, T., & Okada, Y. (2021). HDAC

inhibitor, MS-275, increases vascular permeability by suppressing Robo4 expression in endothelial cells. *Tissue Barriers*, *9*(3), 1911195. https://doi.org/10.1080/21688370.2021.1911195

Kim, H.-C., Choi, K.-C., Choi, H.-K., Kang, H.-B., Kim, M.-J., Lee, Y.-H., Lee, O.-H., Lee, J., Kim, Y. J., Jun, W., Jeong, J.-W., & Yoon, H.-G. (2010). HDAC3 selectively represses CREB3-mediated transcription and migration of metastatic breast cancer cells. *Cellular and Molecular Life Sciences*, *67*(20), 3499–3510. https://doi.org/10.1007/s00018-010-0388-5

Leslie, P. L., Chao, Y. L., Tsai, Y.-H., Ghosh, S. K., Porrello, A., Van Swearingen, A. E. D., Harrison, E. B., Cooley, B. C., Parker, J. S., Carey, L. A., & Pecot, C. V. (2019). Histone deacetylase 11 inhibition promotes breast cancer metastasis from lymph nodes. *Nature Communications*, *10*(1), 4192. https://doi.org/10.1038/s41467-019-12222-5

Li, A., Liu, Z., Li, M., Zhou, S., Xu, Y., Xiao, Y., & Yang, W. (2016). HDAC5, a potential therapeutic target and prognostic biomarker, promotes proliferation, invasion and migration in human breast cancer. *Oncotarget*, *7*(25), 37966–37978. https://doi.org/10.18632/oncotarget.9274

Lindorff-Larsen, K., Piana, S., Palmo, K., Maragakis, P., Klepeis, J. L., Dror, R. O., & Shaw, D. E. (2010). Improved side-chain torsion potentials for the Amber ff99SB protein force field. *Proteins*, *78*(8), 1950–1958. https://doi.org/10.1002/prot.22711

Mottet, D., Bellahcène, A., Pirotte, S., Waltregny, D., Deroanne, C., Lamour, V., Lidereau, R., & Castronovo, V. (2007). Histone Deacetylase 7 Silencing Alters Endothelial Cell Migration, a Key Step in Angiogenesis. *Circulation Research*, *101*(12), 1237–1246. https://doi.org/10.1161/CIRCRESAHA.107.149377

Palma, C. de S., Grassi, M. L., Thomé, C. H., Ferreira, G. A., Albuquerque, D., Pinto, M. T., Ferreira Melo, F. U., Kashima, S., Covas, D. T., Pitteri, S. J., & Faça, V. M. (2016). Proteomic Analysis of Epithelial to Mesenchymal Transition (EMT) Reveals Cross-talk between SNAIL and HDAC1 Proteins in Breast Cancer Cells*. *Molecular & Cellular Proteomics*, *15*(3), 906–917. https://doi.org/10.1074/mcp.M115.052910

Park, S. Y., Jun, J. A., Jeong, K. J., Heo, H. J., Sohn, J. S., Lee, H. Y., Park, C. G., & Kang, J. (2011). Histone deacetylases 1, 6 and 8 are critical for invasion in breast cancer. *Oncology Reports*, *25*(6), 1677–1681. https://doi.org/10.3892/or.2011.1236

Rey, M., Irondelle, M., Waharte, F., Lizarraga, F., & Chavrier, P. (2011). HDAC6 is required for invadopodia activity and invasion by breast tumor cells. *European Journal of Cell Biology*, *90*(2), 128–135. https://doi.org/10.1016/j.ejcb.2010.09.004

Roy, S. S., Gonugunta, V. K., Bandyopadhyay, A., Rao, M. K., Goodall, G. J., Sun, L.-Z., Tekmal, R. R., & Vadlamudi, R. K. (2014). Significance of PELP1/HDAC2/miR-200 regulatory network in EMT and metastasis of breast cancer. *Oncogene*, *33*(28), 3707–3716. https://doi.org/10.1038/onc.2013.332

Salgado, E., Bian, X., Feng, A., Shim, H., & Liang, Z. (2018). Overexpression of HDAC9 Promotes Invasion and Angiogenesis of Triple Negative Breast Cancer by regulating microRNA-206. *Biochemical and Biophysical Research Communications*, *503*(2), 1087–1091. https://doi.org/10.1016/j.bbrc.2018.06.120

Sousa da Silva, A. W., & Vranken, W. F. (2012). ACPYPE - AnteChamber PYthon Parser interfacE. *BMC Research Notes*, *5*(1), 367. https://doi.org/10.1186/1756-0500-5-367

Srivastava, R. K., Kurzrock, R., & Shankar, S. (2010). MS-275 Sensitizes TRAIL-Resistant Breast Cancer Cells, Inhibits Angiogenesis and Metastasis, and Reverses Epithelial-Mesenchymal Transition In vivo. *Molecular Cancer Therapeutics*, *9*(12), 3254–3266. https://doi.org/10.1158/1535-7163.MCT-10-0582

Tang, Z., Ding, S., Huang, H., Luo, P., Qing, B., Zhang, S., & Tang, R. (2017). HDAC1 triggers the proliferation and migration of breast cancer cells via upregulation of interleukin-8. *Biological Chemistry*, *398*(12), 1347–1356. https://doi.org/10.1515/hsz-2017-0155

Urbich, C., Rössig, L., Kaluza, D., Potente, M., Boeckel, J.-N., Knau, A., Diehl, F., Geng, J.-G., Hofmann, W.-K., Zeiher, A. M., & Dimmeler, S. (2009). HDAC5 is a repressor of angiogenesis and determines the angiogenic gene expression pattern of endothelial cells. *Blood*, *113*(22), 5669–5679. https://doi.org/10.1182/blood-2009-01-196485

Vassetti, D., Pagliai, M., & Procacci, P. (2019). Assessment of GAFF2 and OPLS-AA General Force Fields in Combination with the Water Models TIP3P, SPCE, and OPC3 for the Solvation Free Energy of Druglike Organic Molecules. *Journal of Chemical Theory and Computation*, *15*(3), 1983–1995. https://doi.org/10.1021/acs.jctc.8b01039

Yi, Z., Wenwen, L., Kun, W., & Jian, S. (2019). [Overexpression of histone deacetylase 11 suppresses basal-like breast cancer cell invasion and metastasis]. *Nan Fang Yi Ke Da Xue Xue Bao = Journal of Southern Medical University*, *39*(7), 751–759. https://doi.org/10.12122/j.issn.1673-4254.2019.07.01

Zhang, L., Wang, G., Wang, L., Song, C., Leng, Y., Wang, X., & Kang, J. (2012). VPA inhibits breast cancer cell migration by specifically targeting HDAC2 and down-regulating Survivin. *Molecular and Cellular Biochemistry*, *361*(1), 39–45. https://doi.org/10.1007/s11010-011-1085-x

Zheng, X., Wu, Z., Xu, K., Qiu, Y., Su, X., Zhang, Z., & Zhou, M. (2019). Interfering histone deacetylase 4 inhibits the proliferation of vascular smooth muscle cells via regulating MEG3/miR-125a-5p/IRF1. *Cell Adhesion & Migration*, *13*(1), 41–49. https://doi.org/10.1080/19336918.2018.1506653
